# SCALE: unsupervised multiscale domain identification in spatial omics data

**DOI:** 10.1093/nar/gkaf1456

**Published:** 2026-01-06

**Authors:** Behnam Yousefi, Darius P Schaub, Robin Khatri, Nico Kaiser, Malte Kuehl, Cedric Ly, Victor G Puelles, Tobias B Huber, Immo Prinz, Christian F Krebs, Ulf Panzer, Stefan Bonn

**Affiliations:** Institute of Medical Systems Bioinformatics, Center for Biomedical AI (bAIome), Center for Molecular Neurobiology (ZMNH), University Medical Center Hamburg-Eppendorf, Hamburg 20251, Germany; German Center for Child and Adolescent Health (DZKJ), partner site Hamburg, University Medical Center Hamburg-Eppendorf, Hamburg 20251, Germany; Institute of Medical Systems Bioinformatics, Center for Biomedical AI (bAIome), Center for Molecular Neurobiology (ZMNH), University Medical Center Hamburg-Eppendorf, Hamburg 20251, Germany; III. Department of Medicine, University Medical Center Hamburg-Eppendorf, Hamburg 20251, Germany; Institute of Medical Systems Bioinformatics, Center for Biomedical AI (bAIome), Center for Molecular Neurobiology (ZMNH), University Medical Center Hamburg-Eppendorf, Hamburg 20251, Germany; Hamburg Center for Translational Immunology (HCTI), University Medical Center Hamburg-Eppendorf, Hamburg 20251, Germany; Institute of Medical Systems Bioinformatics, Center for Biomedical AI (bAIome), Center for Molecular Neurobiology (ZMNH), University Medical Center Hamburg-Eppendorf, Hamburg 20251, Germany; III. Department of Medicine, University Medical Center Hamburg-Eppendorf, Hamburg 20251, Germany; Department of Clinical Medicine, Aarhus University, Aarhus 8200, Denmark; Department of Pathology, Aarhus University Hospital, Aarhus 8200, Denmark; Institute of Medical Systems Bioinformatics, Center for Biomedical AI (bAIome), Center for Molecular Neurobiology (ZMNH), University Medical Center Hamburg-Eppendorf, Hamburg 20251, Germany; Institute of Systems Immunology, University Medical Center Hamburg-Eppendorf, Hamburg 20251, Germany; III. Department of Medicine, University Medical Center Hamburg-Eppendorf, Hamburg 20251, Germany; Department of Clinical Medicine, Aarhus University, Aarhus 8200, Denmark; Department of Pathology, Aarhus University Hospital, Aarhus 8200, Denmark; Hamburg Center for Kidney Health (HCKH), University Medical Center Hamburg-Eppendorf, Hamburg 20251, Germany; III. Department of Medicine, University Medical Center Hamburg-Eppendorf, Hamburg 20251, Germany; Hamburg Center for Translational Immunology (HCTI), University Medical Center Hamburg-Eppendorf, Hamburg 20251, Germany; Hamburg Center for Kidney Health (HCKH), University Medical Center Hamburg-Eppendorf, Hamburg 20251, Germany; Hamburg Center for Translational Immunology (HCTI), University Medical Center Hamburg-Eppendorf, Hamburg 20251, Germany; Institute of Systems Immunology, University Medical Center Hamburg-Eppendorf, Hamburg 20251, Germany; III. Department of Medicine, University Medical Center Hamburg-Eppendorf, Hamburg 20251, Germany; Hamburg Center for Translational Immunology (HCTI), University Medical Center Hamburg-Eppendorf, Hamburg 20251, Germany; Hamburg Center for Kidney Health (HCKH), University Medical Center Hamburg-Eppendorf, Hamburg 20251, Germany; III. Department of Medicine, University Medical Center Hamburg-Eppendorf, Hamburg 20251, Germany; Hamburg Center for Translational Immunology (HCTI), University Medical Center Hamburg-Eppendorf, Hamburg 20251, Germany; Hamburg Center for Kidney Health (HCKH), University Medical Center Hamburg-Eppendorf, Hamburg 20251, Germany; Institute of Medical Systems Bioinformatics, Center for Biomedical AI (bAIome), Center for Molecular Neurobiology (ZMNH), University Medical Center Hamburg-Eppendorf, Hamburg 20251, Germany; German Center for Child and Adolescent Health (DZKJ), partner site Hamburg, University Medical Center Hamburg-Eppendorf, Hamburg 20251, Germany; Hamburg Center for Translational Immunology (HCTI), University Medical Center Hamburg-Eppendorf, Hamburg 20251, Germany; Hamburg Center for Kidney Health (HCKH), University Medical Center Hamburg-Eppendorf, Hamburg 20251, Germany

## Abstract

Single-cell spatial transcriptomics enables precise mapping of cellular states and functional domains within their native tissue environment. These functional domains often exist at multiple spatial scales, with larger domains encompassing smaller ones, reflecting the hierarchical organization of biological systems. However, the identification of these functional domain hierarchies has been largely unexplored due to the lack of suitable computational methods. In this work, we present SCALE, an unsupervised algorithm for multiscale domain identification in spatial transcriptomics data. SCALE combines deep learning-based graph representation learning with an entropy-based search algorithm to detect functional domains at different scales. We demonstrate its effectiveness in identifying multiscale domains using both simulated data and spatial transcriptomics data from murine brain (Xenium and MERFISH) and patient-derived kidney tissue, highlighting its robustness and scalability across diverse tissue types and platforms. SCALE outperforms state-of-the-art multidomain identification by up to 191.1 percentage points. SCALE’s ease of use makes it a powerful aid for advancing our understanding of tissue organization and function in health and disease.

## Graphical Abstract

**Graphical Abstract:**
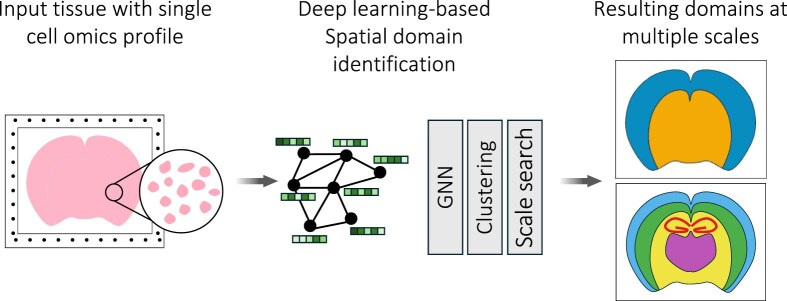

## Introduction

**Figure 1.**
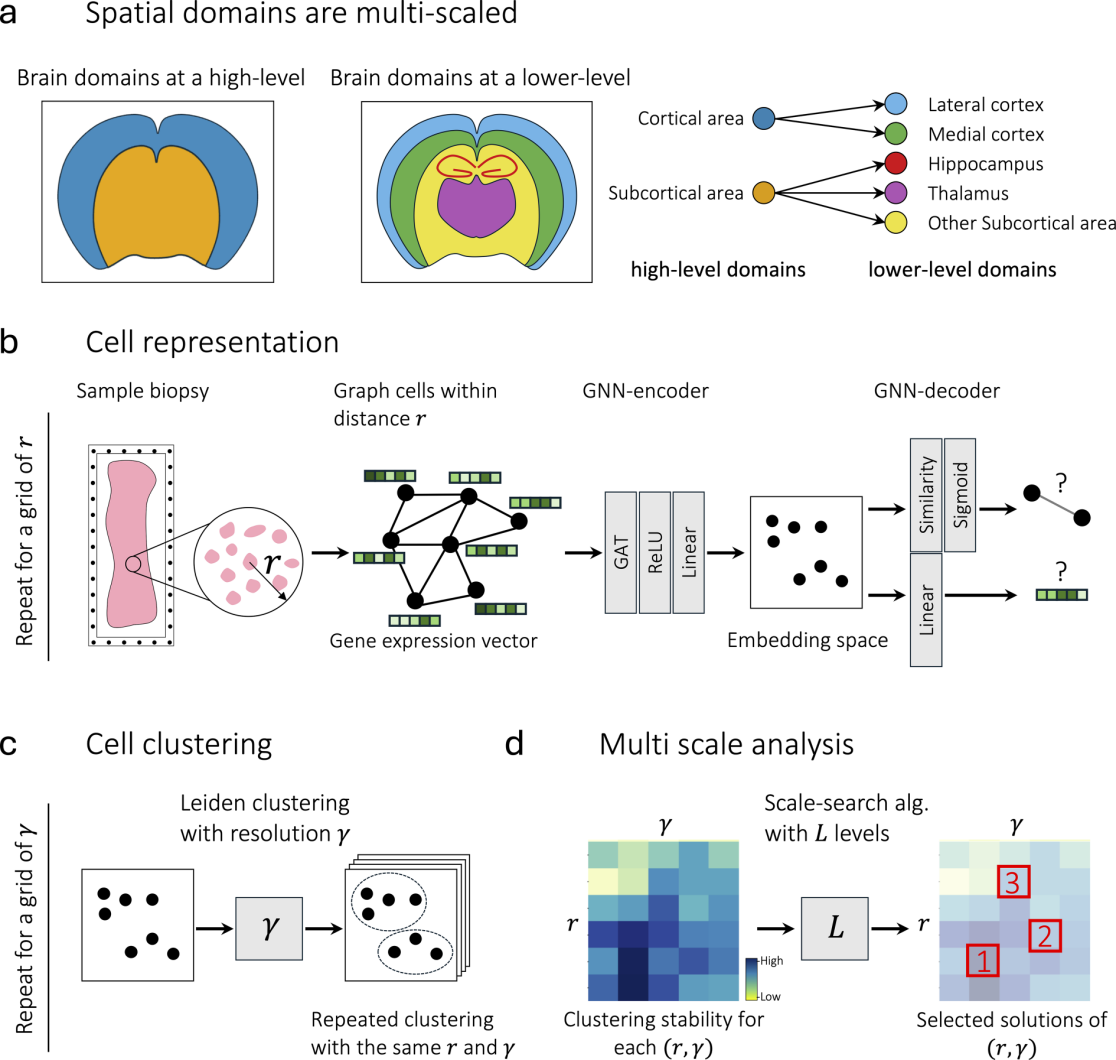
Schematic representation of spatial domain hierarchies and SCALE’s architecture. (**a**) Spatial domains in the brain are organized in a hierarchical, nested structure. (**b**) Given ST data from a tissue biopsy, a spatial graph is constructed where cells are represented as nodes and edges represent cell–cell adjacencies within a threshold $r$. For a grid of different thresholds $r$, a graph neural network-based (GNN-based) encoder–decoder learns cell representations by reconstructing the cellular adjacencies and gene expression. (**c**) Leiden clustering with several resolutions $\gamma $ is used for clustering nodes in embedding space, and a cluster similarity metric measures the stability of clusters generated by each pair of $r$ and $\gamma $. The base assumption is that functional domains should be more stable. (**d**) Our entropy-based search algorithm finds values of $r$ and $\gamma $ for a desired number of levels.

The rapidly growing field of single-cell spatial transcriptomics (ST) enables the identification of cellular states and signaling in their native microenvironments. Within a tissue, cells are often organized into distinct spatially related regions that correspond to specific anatomical structures or functional areas. These anatomical and functional domains exist across multiple scales, often in a nested structure, ranging from local cellular domains to broader anatomical domains (Fig. 1a). Identifying spatial domains across multiple scales can improve our understanding of organ function, development, and disease pathology. However, despite significant advancements in spatial molecular imaging technologies, such as MERFISH [1] and Xenium [2], multiscale domain identification remains an underexplored area, and current methodologies still face substantial challenges in accurately identifying spatial domains, particularly in an unsupervised manner.

Traditional approaches in ST have often adapted clustering algorithms initially designed for single-cell RNA sequencing [3–5], which do not take spatial information into account. Recent works have introduced spatially informed algorithms ranging from GNN-based methods [6–12] to Bayesian methods [13]. Despite these advances, these methods are not designed for capturing multiscale spatial structures. To identify domains across multiple scales, existing methods usually require manual tuning of multiple hyperparameters, while the results may highly depend on the user’s choice. Currently, the only method for the identification of hierarchical domains is NeST [14], which searches regions of highly co-expressed genes. NeST is based on an assumption that domains in a tissue are characterized by specific gene coexpression patterns; however, this assumption may not be generalizable across different tissues, specifically where distinct domains include overlapping cell types, potentially leading to suboptimal performance.

This manuscript introduces SCALE (Spatial Clustering At multiple LEvels), a method that employs a GNN-based encoder–decoder architecture with a bi-objective function integrating both cell transcriptomic data and spatial relationships among cells. GNN representations are subsequently subjected to a novel entropy-based search algorithm that enables the identification of optimal domains across multiple scales. SCALE is easily applicable and identifies spatial domains in simulated and real-world spatial single-cell data obtained from a variety of tissue types and technologies with best-of-breed accuracy and reliability.

## Materials and methods

SCALE employs a GNN-based encoder–decoder architecture to map cells into an embedding space and subsequently applies clustering entangled with a novel entropy-based search algorithm to identify spatial domains. The overall framework of SCALE is illustrated in Fig. 1b–d. Our method is derived from three key assumptions about spatial domain organization: (i) The local coherence assumption states that cells within a spatial domain have similar gene expression in their neighborhood. More intuitively, cells within the same domain tend to be surrounded by a similar composition of cell types. (ii) The spatial continuity and scale relevance assumption states that biological domains form contiguous regions at specific biologically meaningful scales. In other words, cells belonging to the same domain should occupy continuous spatial regions, with this continuity defined relative to the scale at which the tissue is observed. Finally, (iii) the hierarchical organization assumption states that functional regions tend to be organized in a spatially nested manner. While this assumption may not perfectly apply to all tissues, it reflects the general tendency of biological structures to exhibit multiple levels of spatial organization, where larger domains encompass smaller, more specialized subregions. To formalize the first two assumptions, we apply graph representation learning to embed cells into a latent space, such that cells belonging to the same domain are positioned close to one another. Consequently, the third assumption is formalized by applying our entropy-based search algorithm.

### Deep learning-based graph representation learning

Having a spatially resolved sample biopsy, we first construct a spatial graph, with nodes representing cells and their gene expression profiles as node attributes. The edges represent spatial adjacency between cells defined by a spatial distance threshold $r$. In the encoder, a layer of graph attention network [15], followed by a ReLU activation function and a linear layer, is trained to perform message passing between adjacent cells and embed them into an embedding space. The decoder is subsequently trained to reconstruct both the gene expression profiles of the cells and the cell–cell adjacency relationships (Fig. 1b). This approach ensures that, in the embedding space, cells that are spatially close and share similar gene expression neighborhoods will be positioned close to each other. Therefore, to train the model, we proposed to use a bi-objective cost function optimized along a Pareto front as Equation 1,


(1) \begin{eqnarray*}
L = {L_1} + \lambda {L_2},
\end{eqnarray*}

where ${L_1}$ is a mean squared error for gene expression prediction and ${L_2}$ is a binary cross-entropy loss for predicting pairs of cells within a neighborhood. The hyperparameter $\lambda $ enables navigation across the Pareto front, balancing the trade-offs between the objectives (see next). A mathematical description of our deep learning method is presented in the Supplementary File.

### Pareto optimization for $\lambda $

To find optimal trade-offs between spatial and molecular objectives for each $r$, we trained our model with different values of $\lambda $ (Supplementary Table S1), spanning the spectrum from ${L_1}$-dominant to ${L_2}$-dominant. This results in a set of models on the Pareto front. Selecting the best model among those on the Pareto front is not a trivial task as none of the solutions dominate the others. Considering that spatial organization should be preserved (or reflected) in the learned embedding space, we chose the $\lambda $ that maximizes the correlation of the GNN embedding space and spatial adjacencies based on Moran’s I (MI) [using the *morans_i* function from the *Scanpy* (v1.10.1) Python package]. In some cases, the $MI$-$\lambda $ curve reached a plateau, for which we selected the $\lambda $ value corresponding to the saturation point identified by fitting a sigmoid curve (Supplementary Fig. S1). The algorithm is presented in Algorithm 1.

**Algorithm 1.** Algorithm for the optimization of $\lambda $.

**Input:** a set of $R = \{ {{r_1},\; \dots ,{r_n}} \}$, a set of ${\Lambda ^{}} = \{ {\lambda _1^{},\;\dots ,\;\lambda _m^{}} \}\;$ , and GNN embeddings $H_\lambda ^{( r )}$ trained for each $\lambda $ and $r$, a KNN neighborhood graph ($k = 4$) $W = \{ {{w_{ij}}} \}{|_{i\neq j}};\;{w_{ij}}\in \{ {0,1} \}$

**Output:** the set of optimal $\lambda $ values per $r{\mathrm{\;}}$, ${\Lambda ^*} = \{ {\lambda _{}^*[ {{r_1}} ],\;\dots ,\;\lambda _{}^*[ {{r_n}} ]} \}$

1. for $r$ in $R$
2. for $\lambda $ in ${\Lambda}$
3. $m[ \lambda ]$ = Moran’s I ($W$, $H_\lambda ^{( r )}$)
4. s$[ \lambda ]$ = a sigmoid curve fitted on $m[\lambda ]$
5. ${\lambda_{\mathrm{ saturation}}} = {{\mathrm{ arg.min}}_{\lambda}} \;\left\{ {\frac{{{d^2}}}{{d{\lambda ^2}}}s[ \lambda ]} \right\}$
6. ${\lambda _{\mathrm{ peak}}} = {{\mathrm{ arg.max}}_{\lambda}} \;\{ s[\lambda] \}$
7. if $m( {{\lambda _{{\mathrm{ peak}}}}} )\; - m( {{\lambda _{{\mathrm{ saturation}}}}} )\; < 0.05\;.m( {{\lambda _{{\mathrm{ peak}}}}} )$
8. ${\lambda}^{*}[r] = {\lambda}_{{\mathrm{ saturation}}}$ *# pick the saturation point*
9. else
10. $\lambda^*[ r ] = {\lambda _{{\mathrm{ peak}}}}$ *# pick the max value as no saturation is observed*

### Domain identification by clustering

After the model is trained and cells are represented in the embedding space, we apply a hard clustering method to identify spatial domains (Fig. 1c). Here, we use the Leiden clustering algorithm as implemented in the *Scanpy* (v1.10.1) Python package. An important hyperparameter of Leiden is the resolution $\gamma $ that controls the number of clusters.

### Multiscale domain identification by optimizing *r* and $\gamma $

The identified domains largely depend on the choice of $r$ and the Leiden resolution $\gamma $. Selecting these parameters is challenging for two reasons: (i) domain identification is an unsupervised task with no ground truth in real-life scenarios and (ii) there is often no single optimal scale. In reality, however, biological tissues often contain multiple biologically meaningful spatial domains that are organized hierarchically across different scales. To identify an optimized set of $( {r,\gamma } )$ values, we implemented a two-step scale-tuning algorithm approach designed to ensure: (i) cluster stability and (ii) a nested structure of the clusters from different scales (Fig. 1d). These two steps are described in the following.

Cluster stability can be assessed with respect to random initializations of the clustering algorithm or to variations in clustering parameters [16]. Here, we focus on the former, while extending the concept to the latter in the Supplementary File. We start by generating clusters multiple times across a grid of $r$ and $\gamma $ values denoted as $D( {r,\gamma } )$ (Supplementary File, Supplementary Table S1). Then, we assess the stability of the resulting clusters. Among the clusterings obtained from different scales $( {r,\gamma } )$, we select those with larger stability, i.e. being less sensitive to random initializations or variations in resolution [16]. This approach operates on the assumption that more robust clusters are likely to be better aligned with underlying biological structures. We define the stability of clusters generated for each $( {r,\gamma } )$ as Equation 2,


(2) \begin{eqnarray*}
S\left( {r,\gamma } \right) = \mathop \sum \limits_{i \neq j}^{} ARI\left( {{D^{\left( i \right)}}\left( {r,\gamma } \right),{D^{\left( j \right)}}\left( {r,\gamma } \right)} \right),
\end{eqnarray*}

where $ARI$(.) is adjusted rand score (ARI), i.e. a measure used to evaluate the similarity between two data clusterings, implemented by *adjusted_rand_score* function *Scikit-learn* (v1.3.0) Python package. High $S( {r,\gamma } )$ indicates stable domain identification, while low $S( {r,\gamma } )$ suggests the scale $( {r,\gamma } )$ may not be appropriate for domain identification. Calculating the cluster stability allows us to determine a set of the most stable clusterings for the data. We selected the top k (15%) most stable solutions for subsequent analysis, assuming all biologically meaningful domain scales are contained in this set.

Next, we considered that domains identified at lower-level scales will generally be nested within those identified at higher-level scales. Accordingly, we introduced a novel entropy-search algorithm to find sets of $( {r,\gamma } )$ for a desired number of scales, optimizing for a nested structure where lower scale domains align with those at higher scales (Supplementary Fig. S2). The algorithm searches for a pair of domain clusterings ${D_p} = \{ {d_1^p,\;\dots ,d_r^p} \}$ and ${D_q} = \{ {d_1^q,\;\dots ,d_s^q} \}$ at scales $p$ and $q$ that best adhere to the nestedness assumption. We achieve this by minimizing the entropy score described in Equation 3,


(3) \begin{eqnarray*}
H\left( {p,q} \right) = - \frac{1}{s}\mathop \sum \limits_{j = 1}^s \mathop \sum \limits_{i = 1}^r {P_{ij}}lo{g_2}( {{P_{ij}}}),
\end{eqnarray*}

where $i$ and $j$ refer to the index of clusters for levels $p$ and $q$, respectively. ${P_{ij}}$ is the probability that any cell $x$ inside the lower-level cluster $d_j^q$ is also contained in the higher-level cluster $d_i^p$ defined as Equation 4.


(4) \begin{eqnarray*}
{P_{ij}} = P\left(x\in d_i^p|x\in d_j^q\right) = \frac{{\left| {d_i^p\; \cap \;d_j^q} \right|}}{{\left| {d_j^q} \right|}}.
\end{eqnarray*}

This approach naturally extends to $l$ scales by iterating over all possible $l$-tuples of $( {r,\gamma } )$, ordered by the number of clusters. For each $l$-tuple, we compute the entropy score $H$ across all $l - 1$ cluster pairs it contains. The $l$-tuple with the lowest average entropy score is then selected as the final multiscale domain solution. The user can also specify the minimum number of clusters to be added at each scale (we set this to 20 for the mouse brain data, and 5 for the kidney data).

### Evaluation metrics

To evaluate the performance of SCALE in recognizing the “ground-truth” labels and compare it with other methods, we employed three clustering evaluation metrics: *adjusted mutual information* (AMI) score, which quantifies the similarity between the clusterings and the ground-truth labels; *completeness* (COM) score, which quantifies the extent to which all samples with the same ground-truth label are assigned to the same cluster; and *homogeneity* (HOM) score, which quantifies the purity of clusters with respect to the ground-truth labels. All of these metrics were implemented via the *scitkit-learn* (v1.3.0) Python package [17] and are detailed in the Supplementary File.

### Computational resources

Computations were performed on an AMD EPYC 7742 central processing unit (2.25 GHz, 512 MB L3 cache, 64 cores, 128 GB RAM) and an NVIDIA A100 Tensor Core GPU with 40 GB VRAM.

## Data

### Dataset 1

Dataset 1 is a publicly available dataset generated using MERFISH technology [1] by the manufacturer Vizgen. It contains measurements for 483 genes and ~80 000 cells per sample, with cell segmentation performed using Vizgen’s default pipeline. To obtain ground-truth domain annotations, we applied the same workflow as [18] to the four most symmetric samples. Accordingly, we obtained annotations for major brain regions (Level 1) and subregions (Level 2) based on the Allen mouse brain atlas [19].

### Dataset 2

Dataset 2 was produced by 10x Genomics using VisiumHD technology, with a spot size of ~2 μm. It includes one sample of FFPE mouse brain tissue. Instead of using the binned output, we used the segmented output, assigning multiple spots to each segmented cell. We removed all cells containing <100 transcripts and >5000 transcripts, and removed all genes expressed in <25 cells. The processed sample contained 38 890 cells and 14 480 genes. In addition, we followed a similar workflow as for Dataset 1 to obtain ground-truth domain annotations based on the Allen Brain Atlas. Specifically, we relied on a cell density image obtained via rasterization as proposed in the STalign Python library [20] instead of the DAPI image for registration with the Atlas.

### Dataset 3

Dataset 3 is a publicly available dataset generated by 10x Genomics using the Xenium technology with a 5k gene panel. It comprises a single sample of fresh frozen mouse brain tissue from one hemisphere. The data were processed similarly to [18], i.e. we removed all cells containing fewer than 100 transcripts and all genes present in fewer than 25 cells. Moreover, we removed all cells dissociated from the tissue. The processed sample contained 62 047 cells and 4875 genes.

### Dataset 4

Sultana *et al.* [21] generated spatially resolved gene expression data from 64 kidney biopsies. Gene expression was profiled at subcellular resolution using the 10x Xenium platform, employing a custom panel of 480 genes on tissue sections. For pre-processing, we removed cells containing fewer than 10 transcripts and genes expressed in fewer than 5 cells.

### Dataset 5

Dataset 5 is a publicly available dataset comprising three mouse brain samples measured using Xenium technology by 10x Genomics. Each sample includes ~150 000 cells with expression data for 248 unique genes. Cells were segmented using the proprietary pipeline from 10x Genomics. The dataset was recently annotated by Bhuva *et al.* [22] who mapped spatial domains at the transcript level based on the Allen Brain Reference Atlas [19]. The ground-truth annotations for cells and spatial domains were obtained from the original publication [22] available at a single level. We followed the same preprocessing steps as in [18] and excluded dissociated cells by constructing a cell graph using a 60-μm distance threshold and retaining only cells within the largest connected component.

For all datasets, we normalized the raw counts to sum to their medians and applied a log-transformation, respectively, using the *normalize_total* and *log1p* functions of the *Scanpy* (v1.10.1) Python package [3]. Links to the original data are provided in Supplementary Table S2.

## Results

### SCALE can identify domains across multiple scales

In this section, we showcase the performance of SCALE in identifying domains across multiple scales, using different real-world datasets as well as simulated data (Supplementary File). We compare SCALE with NEST, the only other algorithm capable of multiscale domain identification.

### Multiscale analysis of mouse brain data

**Figure 2.**
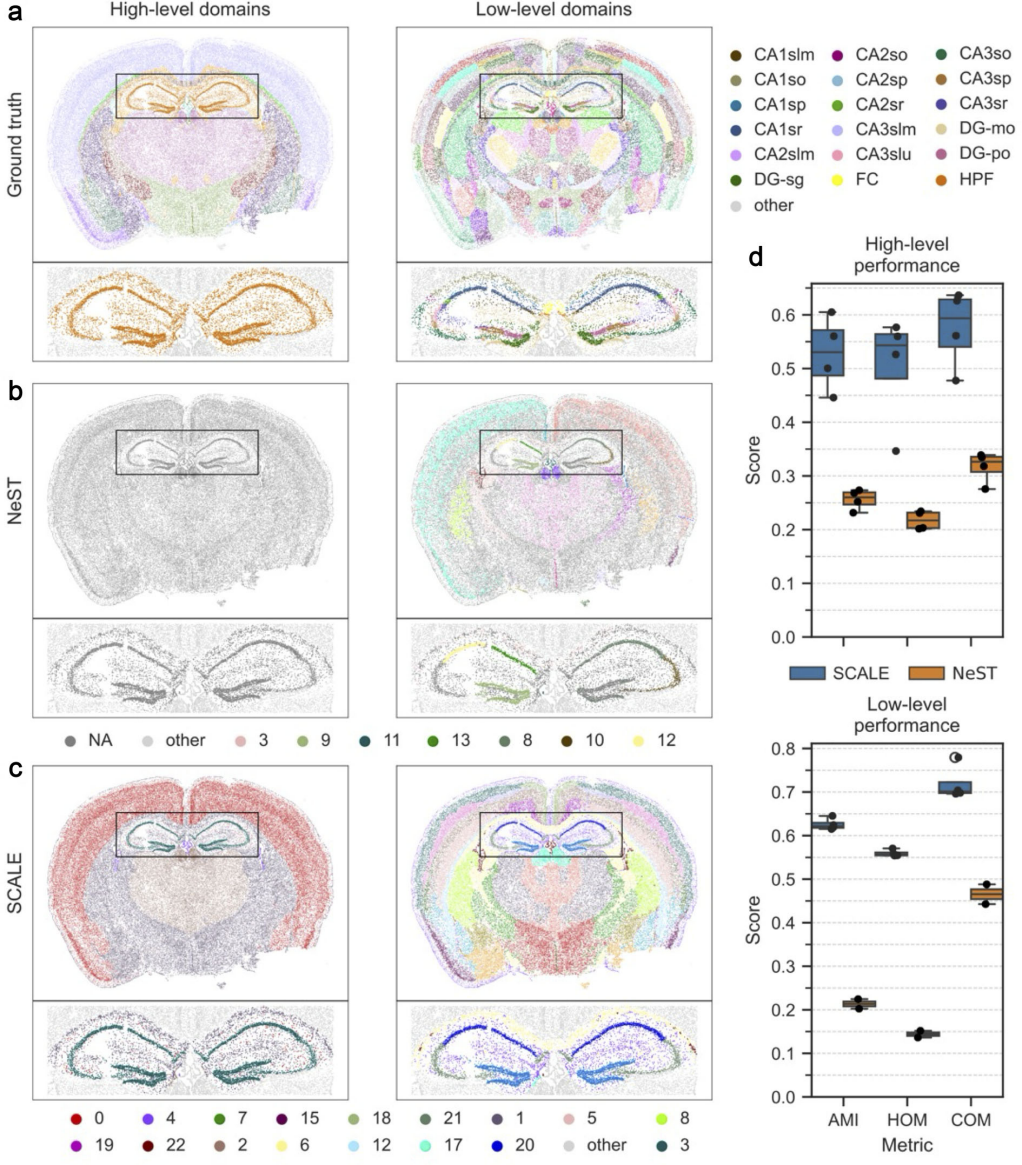
Multiscale domain identification results on MERFISH mouse brain data. (**a**) Ground-truth domain annotations for a selected sample of Dataset 1 were obtained at two levels based on the Allen mouse brain atlas. (**b**) Domains identified by NeST and (**c**) domains identified by SCALE at each scale. The inlets show the domain clusters inside the hippocampal domain; all other cells are grayed out. Cells not assigned to any domain by NeST are labeled as “NA.” Since NeST does not allow the detection of a predefined number of domain levels, it only outputs results for one level on this sample. (**d**) The similarity of identified domains with ground-truth annotations at high- and low-level scales as measured by AMI, HOM, and COM scores. NeST identified domains at two scales only for two of the four samples.

**Figure 3.**
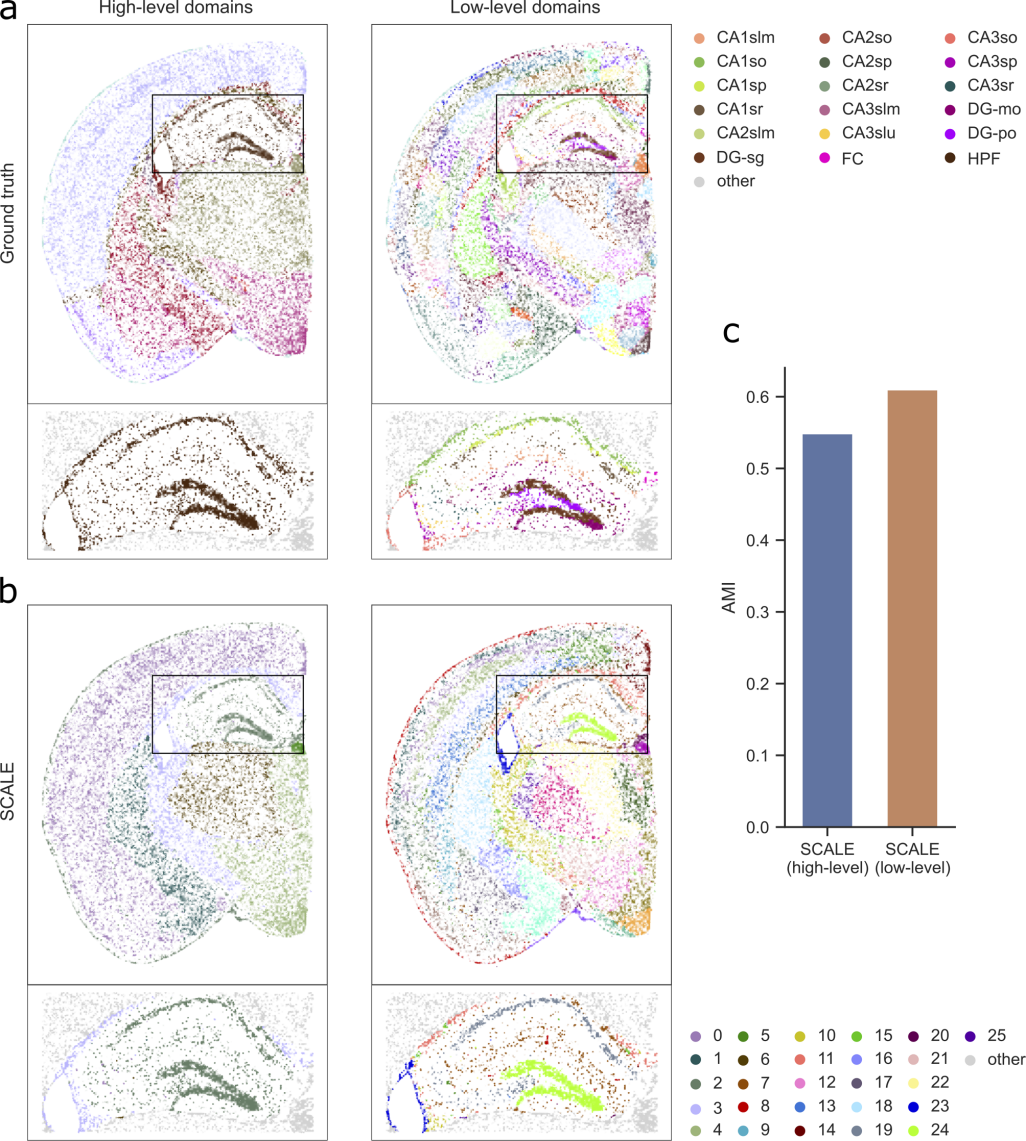
Multiscale domain identification results on Visium–HD mouse brain data. (**a**) Ground-truth domain annotations for a selected sample of Dataset 2 were obtained at two levels based on the Allen mouse brain atlas, and (**b**) domains identified by SCALE at each scale. The inlets show the domain clusters inside the hippocampal domain; all other cells are grayed out. (**c**) The similarity of identified domains with ground-truth annotations at high- and low-level scales as measured by AMI scores.

As an example of real-world data, we used our Datasets 1 and 2, considering both the Level 1 (high-level) and Level 2 (low-level) domains for the ground-truth annotations (Figs 2a and 3a). For Dataset 1, we used NeST and SCALE to identify domains across two scales ($l$ = 2) and three scales ($l$ = 3), with the results presented in Fig. 2 and Supplementary Fig. S3. As can be seen, NeST only detected a single scale with many cells not assigned to any cluster. Moreover, it failed to correctly recognize certain domains, in particular hippocampal subfields. SCALE, on the other hand, showed promising results in identifying multiple scales in the mouse brain data. In particular, as the high-level domains, SCALE identified the brain cortex, hippocampus, and thalamus, which are functionally important areas of the brain. Each of these domains was then divided into lower-level scales such as cortical layers and hippocampal subfields. Figure 2d shows the similarity of the identified domains with the ground-truth annotations at high- and low-level scales. Similar to the above analysis, we used AMI, HOM, and COM scores as the clustering metrics. SCALE demonstrates notably higher similarity to the ground-truth labels at both scales compared to NeST. Specifically, it achieves a median AMI score of 0.53 for high-level domain detection, exceeding NeST by 104.0 percentage points. For low-level domains, SCALE reaches a median AMI score of 0.62, again outperforming NeST by 191.1 percentage points. For Dataset 2, we applied SCALE to identify domains across two scales (l = 2), and we obtained similar results (Fig. 3). As can be seen, SCALE identified the brain cortex, hippocampus, and thalamus at the higher-level domains, while identifying fine-grained substructure at the lower-level domains. SCALE achieves a median AMI scores of 0.55 and 0.62 for high- and low-level domain detection, respectively. In addition, we replaced Leiden with three alternative clustering methods, namely Louvain, k-means, and hierarchical clustering, within our pipeline and found that Leiden consistently outperformed them (Supplementary Figs S5 and S6).

### Multiscale analysis in human kidney data

**Figure 4.**
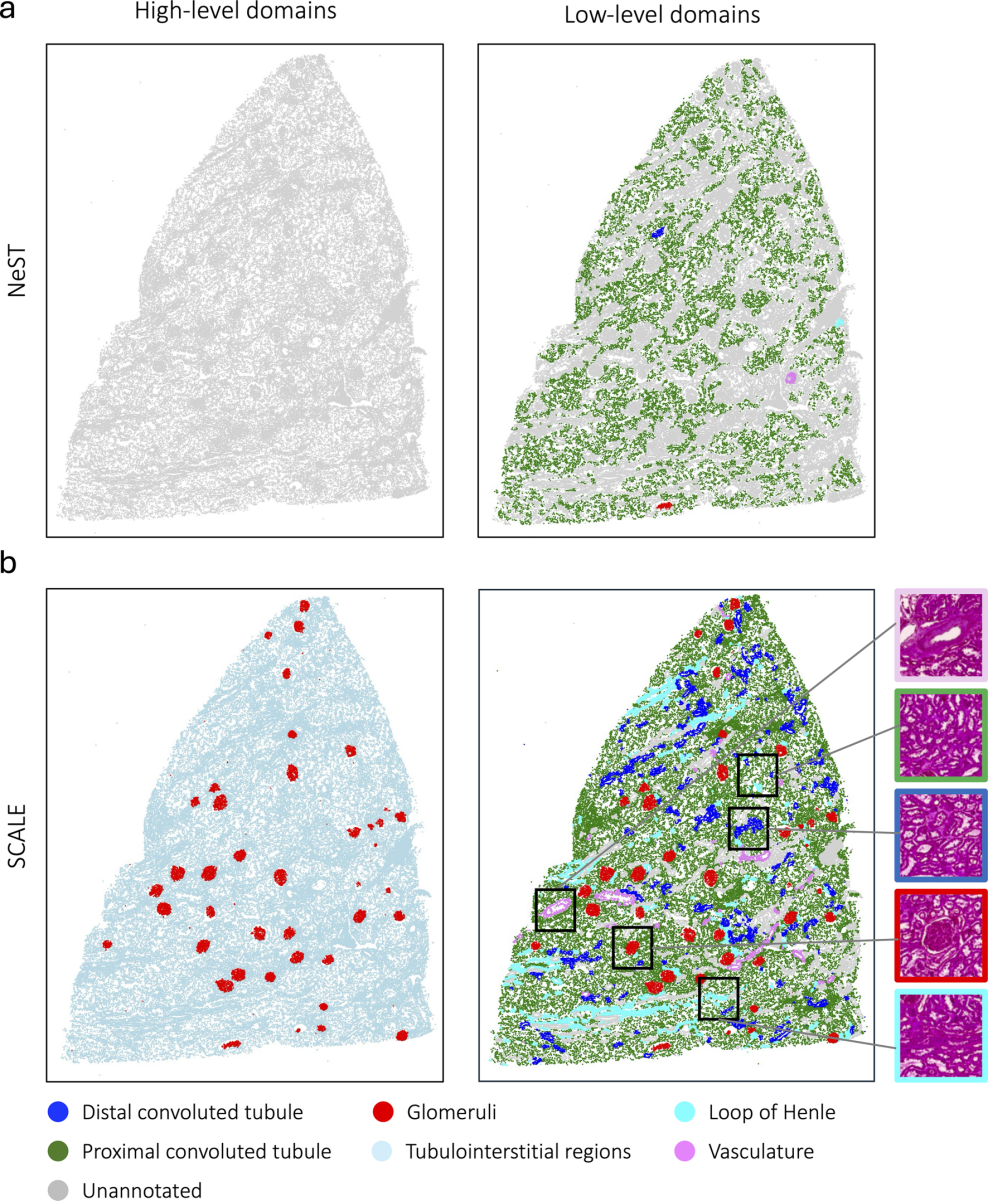
Multiscale domain identification by SCALE and NEST on a human kidney biopsy. All annotations are based on marker genes (e.g. Glomeruli). Domains not assigned to any kidney compartments are denoted as “Unannotated.” (**a**) Domains identified by NeST and (**b**) by SCALE at high- and low-level scales. H&E staining images are provided to showcase kidney compartments. While SCALE reliably captures a high-level representation of glomerular and extra-glomerular regions, NEST fails to find a high-level representation.

We further demonstrate SCALE’s ability to delineate distinct domains in patient-derived kidney biopsies. For this, we used Dataset 4, which consists of gene expression information for a custom gene panel of 480 genes at subcellular resolution measured on tissue sections of the human kidney using the Xenium technology. We employed NeST and SCALE (with $l$ = 2) to identify domains on a control sample (healthy regions of a tumor nephrectomy specimen) shown in Supplementary Fig. S7. Then, we used genetic biomarkers to identify different kidney compartments, including glomeruli, proximal convoluted tube, distal convoluted tube, loop of Henle, and vasculature [23, 24] (Supplementary Fig. S8). Figure 4 shows domains annotated for the higher- and lower-level scales, respectively. NeST only detected a single scale with 58 clusters and a large region of the tissue was not assigned to any cluster, here labeled as “NA” (Supplementary Fig. S3a), which did not align well with the marker genes related to the kidney compartments (Supplementary Fig. S9a). Nevertheless, in the results obtained by SCALE (Fig. 4b), the higher-level scale distinguishes the glomeruli domain from the tubulointerstitial regions; the lower-level scale further identifies finer domains, including proximal convoluted tube, distal convoluted tube, loop of Henle, and vasculature (Supplementary Fig. S9b and c). While comprehensive ground-truth annotations for all kidney domains are generally not feasible, glomerular domains can be annotated by human experts based on H&E staining (Supplementary Fig. S10a). Therefore, we assessed the performance of our method in the recognition of glomerular domains in comparison with expert manual annotations (Supplementary Fig. S10b). Our method demonstrated a sensitivity of 100% and specificity of 88%, showing the capability of SCALE to reliably identify relevant domains in human biopsy data.

### SCALE achieves state-of-the-art single-scale domain identification performance

Since all the existing methods, except for NeST, lack support for multiscale domain identification, we further limited our comparison to single-scale domain identification to ensure a fair evaluation against state-of-the-art methods. For this comparison, we included six methods: NichePCA [18], MENDER [25], Space-Flow [10], SCAN-IT [6], CellCharter [26], Banksy [27], and NeST [14].

**Figure 5.**
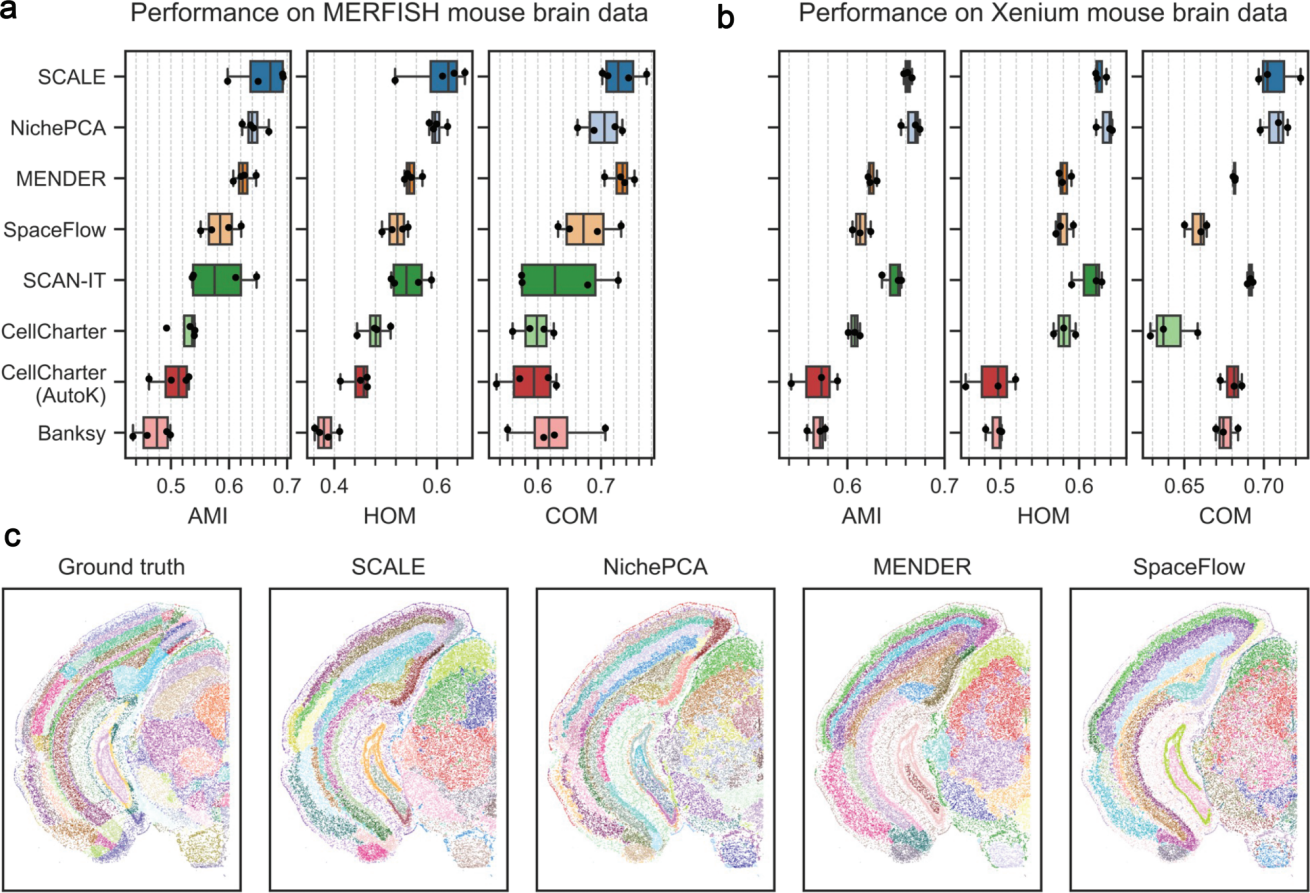
Single-scale domain detection performance comparison on Xenium and MERFISH mouse brain data. (**a**) Benchmarking performance across different methods for MERFISH Dataset 1. Dots show individual sample performance, while the boxplot displays the median, the 25th and 75th percentiles, and whiskers extending 1.5 times the interquartile range. (**b**) Benchmarking performance across different methods for Xenium Dataset 5. (**c**) Example images of the ground-truth domains for the left brain hemisphere of a selected sample of the MERFISH dataset and the identified domains for SCALE, NichePCA, MENDER, and SpaceFlow.

The identification of spatial domains was assessed using the ground-truth annotation based on different cluster similarity metrics. For Dataset 4, we used Level 2 annotations as they show more granular domains. First, we compared SCALE with its automatic hyperparameter selection to competing methods with default parameters. However, all Leiden-based competitors still required supervised tuning of their resolution parameter to optimize for the best match to the ground-truth labels, while SCALE selected it automatically in an unsupervised manner. The only constraint we imposed on SCALE was a minimum of 30 clusters in the selectable clusterings. In this setting, SCALE outperformed all competing algorithms with NichePCA being a close second (Fig. 5a and b, and Supplementary Fig. S11). On Dataset 1, SCALE achieved a median AMI of 0.67, surpassing NichePCA’s, MENDER’s, and NeST’s median AMI by 4.9, 7.6, and 211.7 percentage points, respectively. On Dataset 4, SCALE achieved a median AMI of 0.66, which is almost on par with NichePCA’s AMI of 0.67, while surpassing MENDER’s and NeST’s median AMI by 6.2 and 180.8 percentage points, respectively.

In our next analysis, we tuned all available hyperparameters of the competing methods (e.g. the number of nearest neighbors for constructing the spatial graph) in a supervised manner to achieve the best possible single-scale domain detection performance. In this case, SCALE, although effectively unsupervised with the only constraint that candidate clusterings contain at least 30 clusters, performs on par with NichePCA and outperforms all other methods (Supplementary Fig. S12). The identified spatial domains by different methods, along with their ground-truth annotation for a selected sample of Dataset 1, are shown in Fig. 5c.

In summary, SCALE provides state-of-the-art supervised and unsupervised single-scale domain detection performance, indicating that its GNN-based architecture and dual optimization function are well-suited for the task.

## Discussion

Understanding tissue organization requires the identification of spatial domains and capturing of their hierarchical structure, as biological processes operate across a range of spatial scales—from small, localized niches to broader tissue-level patterns. This hierarchical organization of functional domains is essential for organ function and unsupervised charting of these intricate relationships has to date hardly been investigated. Current domain detection algorithms mostly lack the ability to flexibly detect domains at multiple scales without manual intervention, potentially overlooking important layers of biological organization. In this manuscript, we present SCALE, a deep learning framework designed for the identification of multiscale spatial domains in single-cell ST data. Leveraging a bi-objective training scheme that anchors both transcriptomic similarity and spatial proximity, SCALE learns an embedding space to simultaneously reflect the gene expression neighborhoods of cells and their spatial cellular adjacencies. In addition, SCALE introduces an innovative scale search algorithm that automatically adapts its key parameters $r$ and $\gamma $, enabling robust domain detection across scales with minimal manual calibration. Together, these advances position SCALE as a versatile and scalable tool for decoding tissue organization from spatial omics data.

To implement multiscale domain identification, SCALE employs a two-step solution optimizing procedure for $r$ and $\gamma $: (i) discarding nonrobust solutions and (ii) identifying solutions that best preserve the nested structural organization typically observed in biological tissues. Clusterings that are highly sensitive to random initializations (or variations in resolution) are more likely to reflect noise or local optima rather than meaningful signal, whereas stable solutions indicate the presence of intrinsic structure. Accordingly, SCALE first retains only stable clusterings, among which it then applies an entropy-based algorithm to search for the most optimum nested structures. This combination reduces the chance of artifact-driven clusters and improves the alignment with biological expectations.

To the best of our knowledge, NeST is the only method specifically designed to identify spatial domains across multiple scales. However, in all of our analyses, NeST resulted in suboptimal domain identification performance. In contrast, SCALE showed promising results when tested for multiscale domain identification based on simulated data, mouse brain tissue, and kidney tissue across various technologies, including MERFISH and Xenium (both 500 and 5k panel), as well as Visium-HD. In addition, to show the significance of the scale search algorithm, we tested NichePCA, SCAN-IT, and SpaceFlow on Dataset 1 with low and high resolution, using a naive approach of randomly sampling two Leiden resolutions per sample within the same range as in our SCALE’s search space (Supplementary Figs S13 and S14). These results suggest that naive approaches usually do not provide optimal solutions and might result in discontinuous, ‘shattered’ clusters, as the optimal hyperparameters are tissue dependent.

These results prove SCALE’s versatility across different tissues, ST technologies, and organisms. In the mouse brain tissue, SCALE identified the primary anatomical domains as high-level domains, while simultaneously detecting finer-scale organization, such as cortical layers and hippocampal compartments at a lower level. In the kidney tissue, SCALE annotated glomerular and tubulointerstitial regions as the two high-level domains. At the lower level, SCALE delineated the proximal convoluted tube, the distal convoluted tube, the loop of Henle, and the vasculature as distinct compartments.

We benchmarked SCALE against the state-of-the-art in a single-scale domain identification setting and showed its accuracy and robustness in identifying spatial domains for two independent mouse brain datasets. In particular, we conducted two experiments, both with SCALE optimized in an unsupervised manner. In the first experiment, all methods were run with default parameters, tuning only the Leiden resolution, where SCALE outperformed the competing algorithms. In the second experiment, SCALE was compared against methods optimized using a supervised approach. Although it showed slightly lower performance than NichePCA, it still outperformed or matched all other algorithms. We further demonstrated the fidelity of our automated hyperparameter optimization algorithm by applying it to SCAN-IT, SpaceFlow, and NichePCA (Supplementary Figs S15 and S16). For NichePCA, we used both the KNN-based and distance-based spatial graph construction. While SCALE still demonstrates the best overall performance, NichePCA–KNN and SpaceFlow-KNN, augmented with automatic hyperparameter optimization, show competitive performance. These results suggest that other domain identification algorithms might profit from our newly developed hyperparameter optimization algorithm, especially in multidomain settings. These results demonstrate SCALE’s robustness and adaptability, as it maintains superior performance even when optimized in an unsupervised manner, highlighting its potential for real-world applications where supervised optimization is not feasible.

Despite the superior performance and applicability of SCALE, it does have certain limitations. SCALE is best suited for technologies that offer cell-level resolution (e.g. Xenium and Visium-HD). In contrast, its performance is suboptimal with spot-based platforms that use larger spot sizes, such as Visium, where each spot has a diameter of 50 μm. Larger spots introduce over-smoothing effects that are not well handled by SCALE. While SCALE’s automatic hyperparameter tuning enables effective multiscale domain identification, setting it apart from other methods, it can significantly increase computational time. Users, however, have the flexibility to bypass our hyperparameter tuning and input their own parameters. Future work could focus on enhancing the efficiency of this process by integrating faster search algorithms, such as evolutionary search methods, to improve the scalability and speed of SCALE further. We also acknowledge that some low-level domains, such as vasculature, may span multiple high-level domains, making it difficult for SCALE to capture them as coherent units. This hierarchical overlap may lead to the exclusion of biologically meaningful structures that are not confined to a single higher-level domain. Future work will be necessary to better handle these corner cases.

## Conclusions

Overall, SCALE represents a significant advancement in unsupervised, multiscale spatial domain identification by leveraging GNNs and information theory to uncover nested functional structures within ST data. Unlike existing methods, SCALE reliably detects hierarchical domains across diverse tissue types and technologies, demonstrating superior performance in both simulated and real-world datasets. Its ability to generalize across different spatial omics platforms and tissue types underscores its adaptability and real-world applicability. SCALE provides a powerful framework for advancing our understanding of tissue organization in both health and disease, with potential implications for precision medicine and clinical decision-making.

## Supplementary Material

gkaf1456_Supplemental_Files

## Data Availability

All the raw data can be downloaded from the links provided in Supplementary Table S2. The workflows for further processing and domain annotation are described in the “Materials and methods” section. The code for SCALE is available at https://github.com/imsb-uke/scale (DOI: 10.5281/zenodo.17803419), providing all components necessary to reproduce the analyses.
