# Supplementary material for: SCALE: unsupervised multiscale domain identification in spatial omics data
Source: Nucleic Acids Res. 2026 Jan 6;54(1):gkaf1456. doi: 10.1093/nar/gkaf1456 (PMC12774663; doi:10.1093/nar/gkaf1456)
Supplement: gkaf1456_Supplemental_Files [file gkaf1456_supplemental_files.zip › Yousefi et al supplementary materials 1.pdf]

**Supplementary File 1**

# SCALE: Unsupervised Multi-Scale Domain Identification in Spatial Omics Data

Behnam Yousefi[1,2,*], Darius P. Schaub[1,3,*], Robin Khatri[1,8], Nico Kaiser[1,3], Malte Kuehl[6,7], Cedric Ly[1,4], Victor G. Puelles[3,5,6,7], Tobias B. Huber[3,5,8], Immo Prinz[4,8], Christian F. Krebs[3,5,8], Ulf Panzer[3,5,8], Stefan Bonn[1,2,5,8]

[1] Institute of Medical Systems Bioinformatics, Center for Biomedical AI (bAIome), Center for Molecular Neurobiology (ZMNH), University Medical Center Hamburg-Eppendorf, Hamburg, 20251, Germany.

[2] German Center for Child and Adolescent Health (DZKJ), partner site Hamburg, University Medical Center Hamburg-Eppendorf, Germany.

[3] III. Department of Medicine, University Medical Center Hamburg-Eppendorf, Hamburg, Germany.

[4] Institute of Systems Immunology, University Medical Center Hamburg-Eppendorf, Hamburg, Germany

[5] Hamburg Center for Kidney Health (HCKH), University Medical Center Hamburg-Eppendorf, Hamburg, 20251, Germany.

[6] Department of Clinical Medicine, Aarhus University, Aarhus, Denmark.

[7] Department of Pathology, Aarhus University Hospital, Aarhus, Denmark.

[8] Hamburg Center for Translational Immunology (HCTI), University Medical Center Hamburg-Eppendorf, Hamburg, 20251, Germany.

*equal contribution

Correspondence: Behnam Yousefi (behnam.yousefi@zmnh.uni-hamburg.de), Stefan Bonn (sbonn@uke.de)

# Supplementary Methods

## Mathematical description of SCALE

Given a set of $n$ cells $C = \{c_1, \dots, c_n\}$ in a tissue, each with $s$ dimensional spatial coordinates $y_i \in R^s$ and $g$ dimensional gene expression profiles $x_i \in R^g$, we developed a SCALE to identify spatial domains $D_p = \{d_1^p, \dots, d_k^p\}$ at scale $p$, where the number of domains $k$ is unknown a priori. Each domain $d_i^p$ is a subset of $C$ such that $d_i \cap d_j = \emptyset; \quad \forall\, i \neq j$.

### Assumptions

Our approach is derived from three minimal assumptions about the nature of spatial domains in biological tissues:

1. Local coherence: cells in a spatial domain have similar gene expression profiles in their neighborhood.
2. Spatial continuity and scale relevance: biological domains form spatially contiguous regions, with their structure being most pronounced at specific biologically meaningful scales.
3. Hierarchical organization: functional domains generally exhibit a spatially nested structure.

To satisfy our assumptions, we first defined a spatial graph and then used GNN-based representation learning to embed cells into a vector space. We introduced a bi-objective function to integrate both the gene expression similarity and spatial proximity representation while training the model. We then presented a scale-tuning algorithm to identify domains at multiple nested tissue scales.

### Representing spatial proximity as a spatial graph

Let $G_r = G(V, E_r, X)$ be a graph with the sets of nodes (vertices) $V = \{v_i\}$, edges $E_r = \{e^{(r)}{}_{ij}\}$, and node features $X \in R^{n \times g}$. To define a spatial graph, we let $V = C$ represent the cells as nodes and $X$ denote the expression profiles of cells. $E_r$ represents spatial proximity within the distance threshold $r$. In particular, the existence of an edge $e^{(r)}{}_{ij}$ between cells $c_i$ and $c_j$ is determined by Equitation S1

$$e^{(r)}{}_{ij} \in E \Leftrightarrow \| x_i - x_j \| \leq r \quad \text{(S1)}$$

**GNN-based representation learning**

While several architectures can be used to incorporate our assumptions, a GNN-based model is naturally suitable as it inherently takes into account spatial proximity as a graph and is flexible to ensure local coherence. We defined the model architecture below:

Let $h$ be the dimension of the learned embedding space $Z \in R^{n\times h}$. The network architecture consists of an encoder for graph representation learning, a link prediction decoder, and a gene expression decoder. The encoder learns a mapping function $f_\theta$ that maps $G_r$ into the embedding space $Z_r$ (Equation S2),

$$f_\theta = f_{linear} \circ f_{ReLU} \circ f_{GAT} : R^{n\times(n+g)} \rightarrow R^{n\times h}, \quad \text{(S2)}$$

The link prediction decoder maps the embeddings of a pair of cells onto a scalar value as a probability representing the existence of an edge (Equation S3),

$$g_\phi = g_{Sigmoid} \circ g_{linear} \circ g_{sim}: R^{n^2\times h} \rightarrow R^{n^2\times 1} \quad \text{(S3)}$$

where $g_{sim}(i,j) = -||f_\theta(i) - f_\theta(j)||^2 \ \ \forall i,j \ \ s.d. e_{ij} \in E$ . Finally, the gene expression decoder maps the embedding of each cell onto a reconstructed gene expression vector (Equation S4).

$$h_\psi = h_{linear}: R^{n\times h} \rightarrow R^{n\times g} \quad \text{(S4)}$$

**Objective function**

We defined a bi-objective function that incorporates both decoders of spatial and molecular objectives as Equation S5,

$$L(\theta, \phi, \psi) = L_1(\theta, \phi, \psi) + \lambda L_2(\theta, \phi, \psi) \quad \text{(S5)}$$

where:

- $L_1 = BCE\big(g_\phi(f_\theta(G_r)), A\big)$ is the binary cross-entropy (BCE) loss for link prediction, encouraging the model to capture local spatial structure.

- $L_2 = MSE(h_\psi(f_\theta(G_r)), Y)$ is the mean squared error (MSE) for gene expression prediction, ensuring the embeddings capture global gene expression patterns.

**Domain identification by clustering**

For a given radius $r$, we constructed $G_r$ and computed the embeddings $Z_r = f_\theta(G_r)$. Next, we applied a hard clustering method on $Z_r$ to identify spatial domains satisfying $d_i \cap d_j = \emptyset;\ \forall\, i \neq j$. Here, we used the Leiden clustering algorithm as implemented in the *Scanpy* (v1.10.1) Python package. An important hyperparameter of Leiden is the resolution $\gamma$ that controls the number of clusters. Hence, the final clustering $D$ is a function of $(r, \gamma)$.

## Cluster Stability Analysis

Cluster stability can be assessed based on the stability against random initializations of the clustering algorithm or variations in clustering parameters. A method based on the former was developed in the main text. However, not all clustering algorithms depend on random initializations. Extending the concept to the latter, we developed a new version of our stability that is compatible with deterministic clustering methods. In particular, for a given $r$ and $\gamma$ instead of calculating similarity between clustering results resulting from different seeds, we calculate the similarity to clusterings for $r - \delta$ and $r + \delta$ but the same $\gamma$ as Equation S6.

$$S(r, \gamma) = \sum_{r\prime = \{r-\delta, r, r+\delta\}} ARI(D(r', \gamma), D(r', \gamma)) \tag{S6}$$

## Ranges for $r$ and $\gamma$

To perform our scale search algorithm, we need to define a range $r$ and $\gamma$, which can slightly vary based on the size of the cells in the tissue of interest and the number of expected domains. In our analysis, the minimum range for $r$ is set to 15μm, since smaller values would largely capture subcellular scales and the neighborhood would not extend beyond a single cell. The maximum range for $r$ is set to 55μm, which corresponds to a neighborhood encompassing ~20 cells, the typical distance over which cells can influence each other through paracrine signaling. The choice for $\gamma$ depends on the number of expected clusters. In our analysis, $\gamma$ is restricted to 0.01 to 1.2 that covers cluster numbers of 1 to >60. The user, however, can opt for testing larger values for $r$ and

$\gamma$ based on the biological insight for their tissue of interest. The summary of the hyperparameters is presented in Supplementary Table 1.

## Spatially variable genes

To improve the robustness and performance of SCALE on data with a high number of genes, such as Xenium 5k or VisiumHD, we added a feature selection step. To this end, we calculate spatially variable genes using Moran's I as implemented in the Squidpy package (1) based on a spatial graph with a 40-µm distance cutoff. This function determines the significance level by comparing the observed Moran's I value with the empirical distribution generated by calculating Moran's I for random permutations of the gene expression across cells. Finally, we select the 500 significantly spatially variable genes with the highest Moran's I. This approach has been previously found to perform on par with other, more sophisticated and computationally demanding methods (2). However, SCALE also supports spatially variable genes calculated with other methods.

## Evaluation metrics

*Adjusted mutual information score* (AMI): AMI quantifies the similarity between two clusterings $U$ and $V$ (i.e. in our case, the model-generated clusters and the ground truth) based on their mutual information $MI(U, V)$ while accounting for the possibility that some agreement between clusterings could happen randomly. AMI is defined in Equation S7,

$$AMI(U, V) = \frac{MI(U,V) - E(MI(U,V))}{avg(H(U),H(V)) - E(MI(U,V))} \tag{S7}$$

where $MI()$, $E()$ and $avg()$ represent the mutual information, expectation, and average, respectively. The score is normalized, with a value of 1 indicating perfect clustering correspondence, a value of 0 indicating that the similarity is no better than random, and negative values showing worse than random correspondence.

*Homogeneity score* (HOM): HOM is a score evaluating the purity of clusters with respect to the ground-truth labels. It measures whether each cluster primarily contains data points belonging to a single class defined in Equation S8,

$$HOM(U, V) \ = 1 - \frac{H(U|V)}{H(U)} \tag{S8}$$

where $H()$ is the Shannon entropy. A HOM of 1 indicates perfect homogeneity, meaning each cluster is entirely composed of a single class, while a score of 0 indicates poor homogeneity.

*Completeness score* (COM): COM is a score evaluating the extent to which all data points belonging to a given ground-truth label are assigned to the same cluster recognized by the models defined in Equation S9,

$$HOM(U,V) = 1 - \frac{H(V|U)}{H(V)} \tag{S9}$$

A COM of 1 indicates perfect completeness, meaning all members of each class are grouped within a single cluster, while a score of 0 suggests that members of the same class are distributed across multiple clusters.

## Benchmarking workflow

We used the ground truth annotations for Datasets 1 and 2 to quantitatively compare the performance of SCALE with the state-of-the-art. We additionally attempted to compare our method against STAGATE (3), BASS (4), SpatialPCA (5), and SpaGCN (6), but we were not able to execute them on either Dataset within a memory budget of 128 GB. For SCALE, we applied our automatic cluster-stability-based search procedure per sample to determine the distance cutoff for spatial graph construction and the resolution for Leiden clustering (with the only restriction that the number of clusters should be at least 30). No other method allows for this kind of unsupervised parameter selection. CellCharter offers a similar procedure, called AutoK, but it only determines the number of clusters. For their algorithm, we limited the number of clusters to be between 20 and 60. To make the results for the existing methods comparable, we selected the default distance cutoff for graph construction (or similar parameters, depending on the method) while choosing the cluster resolution (or number of clusters) in a supervised fashion per sample, i.e., we selected the resolution resulting in the best AMI score. Note that this comparison favors the competing methods over SCALE.

Moreover, we compared SCALE against competing methods with tuned hyperparameters, i.e., not only the resolution (or number of clusters) but also another method-specific parameter was selected to maximize the AMI score. The parameter space for each method is provided in Supplementary Table 3.

## Multi-scale analysis in simulated data.

To simulate spatial single-cell data at multiple scales, we manually crafted an image of spatial domains with predefined structures at two levels. We then randomly sampled 1,000 points uniformly across the image representing the cell coordinates (Supplementary Figure 17). The image was structured to contain two large-scale domains, each subdivided into two smaller-scale clusters, thereby capturing both higher- and lower-level spatial organization. A gene expression vector was assigned to each cell using the mouse brain data presented in Moffitt et al. (7). At the finest scale, we considered each domain to be composed of a homogeneous mixture of randomly picked cell types. Details of the algorithm used to assign gene expression profiles to individual cells are provided in Supplementary Algorithm I.

We applied both NeST (8) and SCALE (with two levels, $l=2$) to the simulated data to identify spatial domains (Figure 2b, c). As can be seen, the domains identified by SCALE align almost perfectly with the ground truth labels, while NeST fails to recognize the domains correctly and leaves most cells unassigned. For a quantitative comparison of the predicted with the ground truth domains, we used AMI, HOM, and COM scores. As shown in Figure 2d, SCALE reliably identifies the ground truth domains at different scales, whereas NeST performs poorly. For high-level domain identification, SCALE achieves a median AMI score of 0.85, surpassing NeST by 158.4 percentage points. In the low-level case, SCALE reaches an AMI score of 0.75, again outperforming NeST by 42.5 percentage points.

## Comparison of SCALE to similar methods

Among all the methods we used for comparison, NeST (8) is the only method specifically designed to identify spatial domains across multiple scales. Building on the assumption that spatial domains are characterized by gene co-expression patterns, NeST identifies contiguous subsets of spots or cells that share highly co-expressed genes. Since these patterns may occur at multiple, overlapping scales, NeST can capture spatial domains at varying resolutions. However, when such patterns are absent or not detectable, the resulting spatial domains tend to be sparse and not accurate. In contrast, SCALE is designed based on the assumptions of local coherence, spatial continuity, and hierarchical organization. Accordingly, SCALE constructs a spatial graph of cells and learns a deep learning–based cell representation space reflecting the first two assumptions. Then, a scale

search algorithm is applied, satisfying the third assumption. Notably, SCAN-IT (9) and SpaceFlow (10) share a comparable architecture for cell representation learning, yet do not explicitly target multi-scale domain identification. Both adopt the deep graph infomax (DGI) framework, in which a GNN is trained to distinguish whether a node belongs to the original graph or to a corrupted version. Conceptually, DGI brings nodes closer together if their global roles are similar. SCALE, however, uses a different objective; it jointly optimizes link prediction loss and expression reconstruction loss. The first encourages nodes to be close in the embedding space if they are geometrically close, while the second does so if they have similar local expression patterns. These two complementary losses directly align with our initial assumptions, resulting in better performance in domain identification.

## Runtime and memory analysis

We analyze the runtime and memory complexity empirically by running SCALE on simulated samples containing 20,000, 40,000, 60,000, 80,000, and 100,000 cells, respectively. The data was simulated based on the VisiumHD data, thus containing 14,480 genes. For each sample, we ran SCALE combined with the spatially variable gene selection step (Supplementary Figure 18). It is worth noting that the computational complexity of our algorithm scales linearly with the number of genes, as the first linear layer in the GNN projects the data into a low-dimensional embedding space.

## Domain annotation based on marker genes

After domain identification, we annotated the kidney compartments using the compartment-specific marker gene sets (Supplementary Figure 8). For each gene set, we calculated a score by computing the average expression of its constituent genes. These scores were averaged across all cells in a domain to obtain domain-specific kidney compartment scores. Finally, we assigned the kidney compartment with the highest score to a domain (Supplementary Figure 9).

## References for supplementary file

1. Palla, Giovanni, et al. "Squidpy: a scalable framework for spatial omics analysis." Nature methods 19.2 (2022): 171-178.

2. Li, Zhijian, Zain M. Patel, and Dongyuan Song. "Benchmarking computational methods to identify spatially variable genes and peaks. bioRxiv." Published online (2023).

3. Dong, Kangning, and Shihua Zhang. "Deciphering spatial domains from spatially resolved transcriptomics with an adaptive graph attention auto-encoder." Nature communications 13.1 (2022): 1739.

4. Li, Zheng, and Xiang Zhou. "BASS: multi-scale and multi-sample analysis enables accurate cell type clustering and spatial domain detection in spatial transcriptomic studies." Genome biology 23.1 (2022): 168.

5. Shang, Lulu, and Xiang Zhou. "Spatially aware dimension reduction for spatial transcriptomics." Nature communications 13.1 (2022): 7203.

6. Hu, Jian, et al. "SpaGCN: Integrating gene expression, spatial location and histology to identify spatial domains and spatially variable genes by graph convolutional network." Nature methods 18.11 (2021): 1342-1351.

7. Moffitt, Jeffrey R., et al. "Molecular, spatial, and functional single-cell profiling of the hypothalamic preoptic region." Science 362.6416 (2018): eaau5324.

8. Walker, Benjamin L., and Qing Nie. "NeST: nested hierarchical structure identification in spatial transcriptomic data." Nature communications 14.1 (2023): 6554.

9. Cang, Zixuan, et al. "SCAN-IT: Domain segmentation of spatial transcriptomics images by graph neural network." BMVC: proceedings of the british machine vision conference. british machine vision conference. Vol. 32. 2021.

10. Ren, Honglei, et al. "Identifying multicellular spatiotemporal organization of cells with SpaceFlow." Nature communications 13.1 (2022): 4076.

# Supplementary Algorithms

**Supplementary Algorithm I.** Algorithm for the assignment of gene expression values to cells in the simulated data.

**Input:** a reference gene expression $X$ ($cell \times expression$) along with the vector of corresponding cell types $T$. A set of cells $C$ with their domains $D$ at the finest scale. Noise probability $p_{noise}$

**Output:** gene expression matrix $Y$ for cells in $C$

for $d$ in $D$

    $type_1[d]$, $type_2[d]$ = randomly choose two cell types without replacement $\subset T$

for $i$ in $C$

    $d = D[i]$

    $v$ = random variable $\sim uniform(0, 1)$

    if $v > p_{noise}$

        $t$ = randomly choose a cell type $\in \{type_1[d], type_2[d]\}$

    else

        $t$ = randomly choose a cell type $\notin \{type_1[d], type_2[d]\}$

    $cell$ = randomly select a cell of type $t$ from $T$

    $Y[i,:]$ = m $X[cell,:]$

# Supplementary Figures

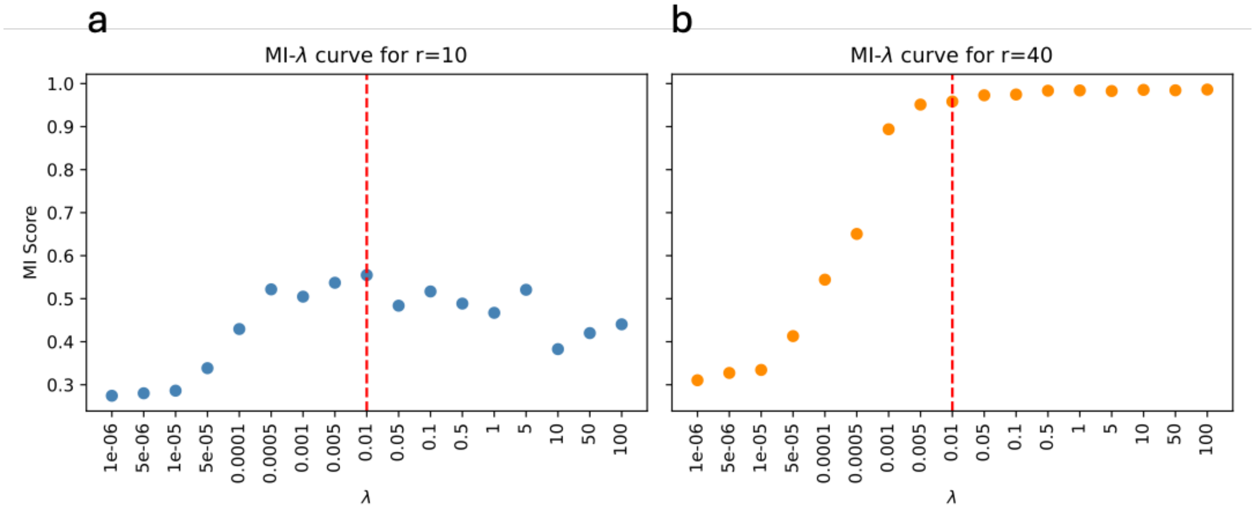


**Supplementary Figure 1.** Two instances of Moran'I (MI)-$\lambda$ curves for a sample from Dataset 1. **(a)** at $r = 10$, MI reaches a peak at $\lambda = 0.01$; while **(b)** at $r = 40$, MI-$\lambda$ shows a saturation at $\lambda = 0.01$. The dashed line indicated the optimum λ.

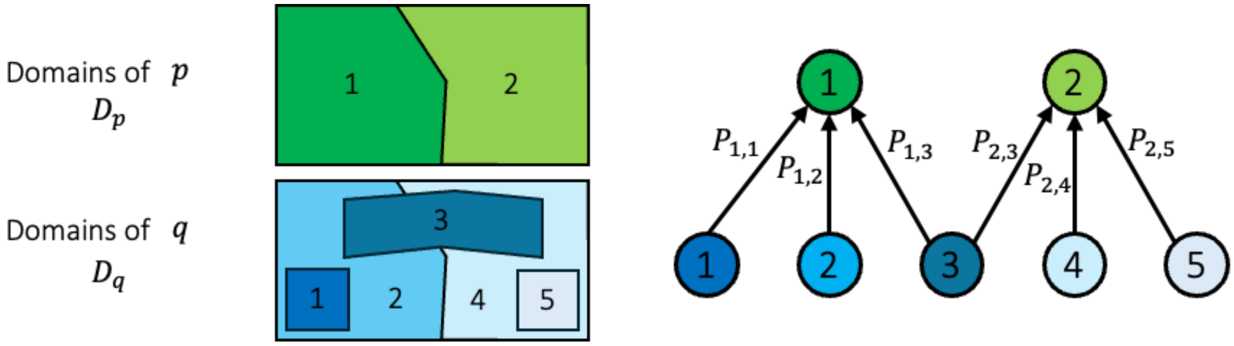


**Supplementary Figure 2.** An example representing the entropy-based search algorithm. **(a)** sets of spatial clusters $D_p = \{d_1^p, d_2^p\}$ (shown in different shades of green) and $D_q = \{d_1^q, \ldots, d_5^q\}$ (shown in different shades of blue) corresponding to two different solutions $p$ and $q$. **(b)** Probability values $P_{ij} = P(x \in d_i^p | x \in d_j^q)$ can be represented as a tree. In this example, Cluster 3 at $q$ is shared between the two clusters at $p$ disrupting the nested structure and leading to an undesirable solution.

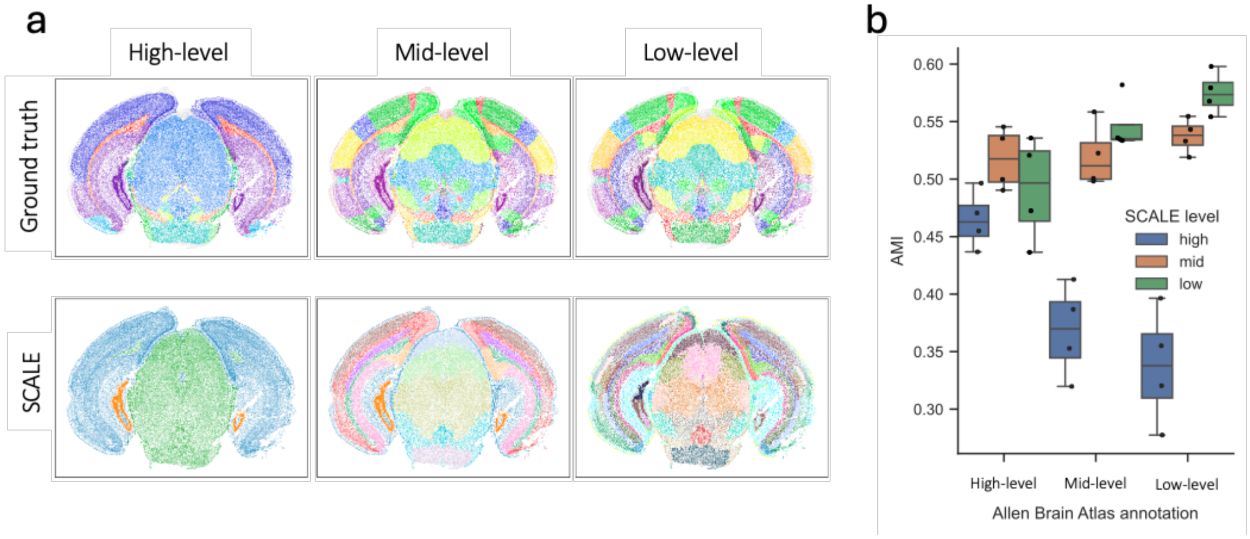


**Supplementary Figure 3.** SCALE domain identification results for three distinct scales on Dataset 1. **(a)** an example of identified domains and their ground-truth annotations based on the Allen Brain Atlas. **(b)** Quantitative performance evaluation in terms of AMI compared to the ground-truth annotations.

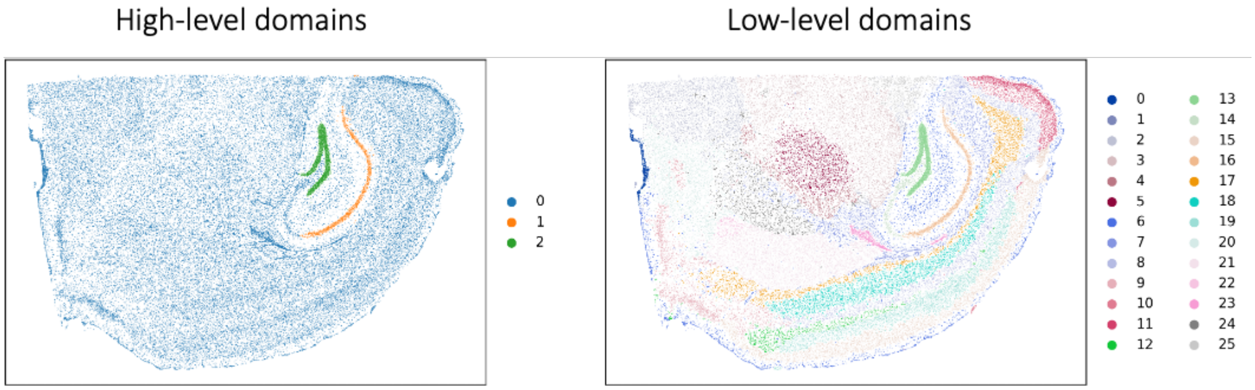


**Supplementary Figure 4.** High-level and low-level domains identified for Dataset 3.

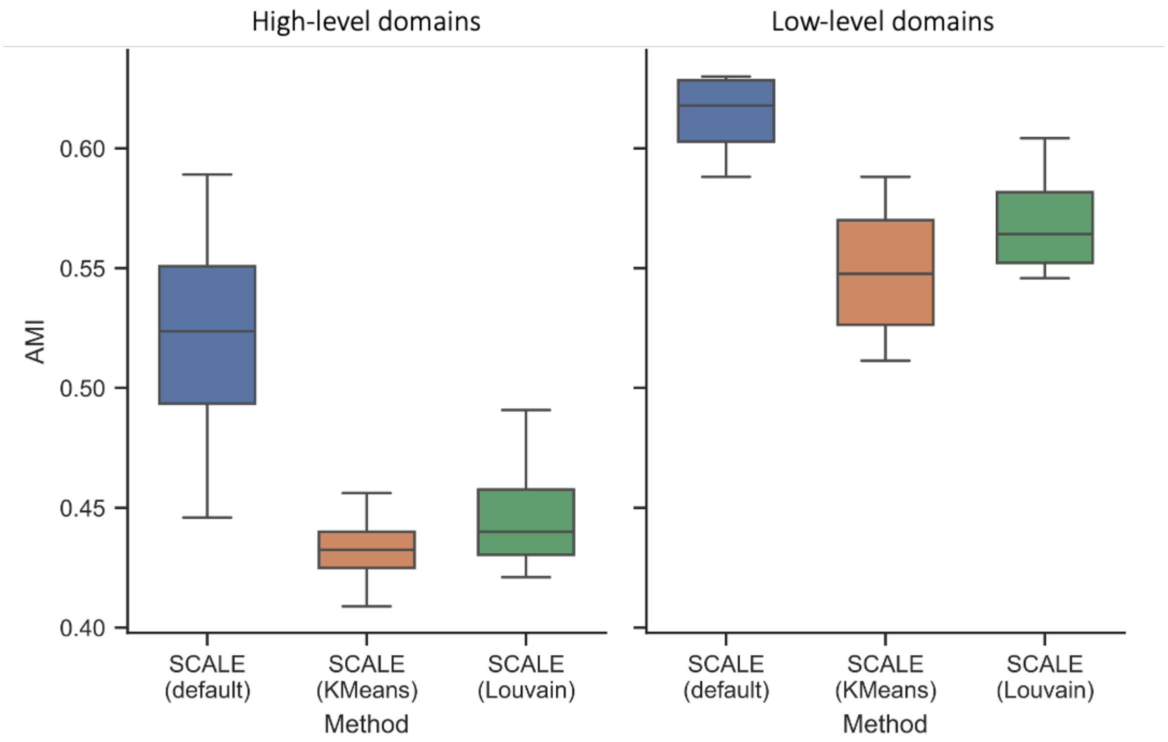


**Supplementary Figure 5.** Performance comparison of SCALE with different clustering algorithms (Leiden, Louvain, and k-means) on the Merfish mouse brain dataset in terms of AMI.

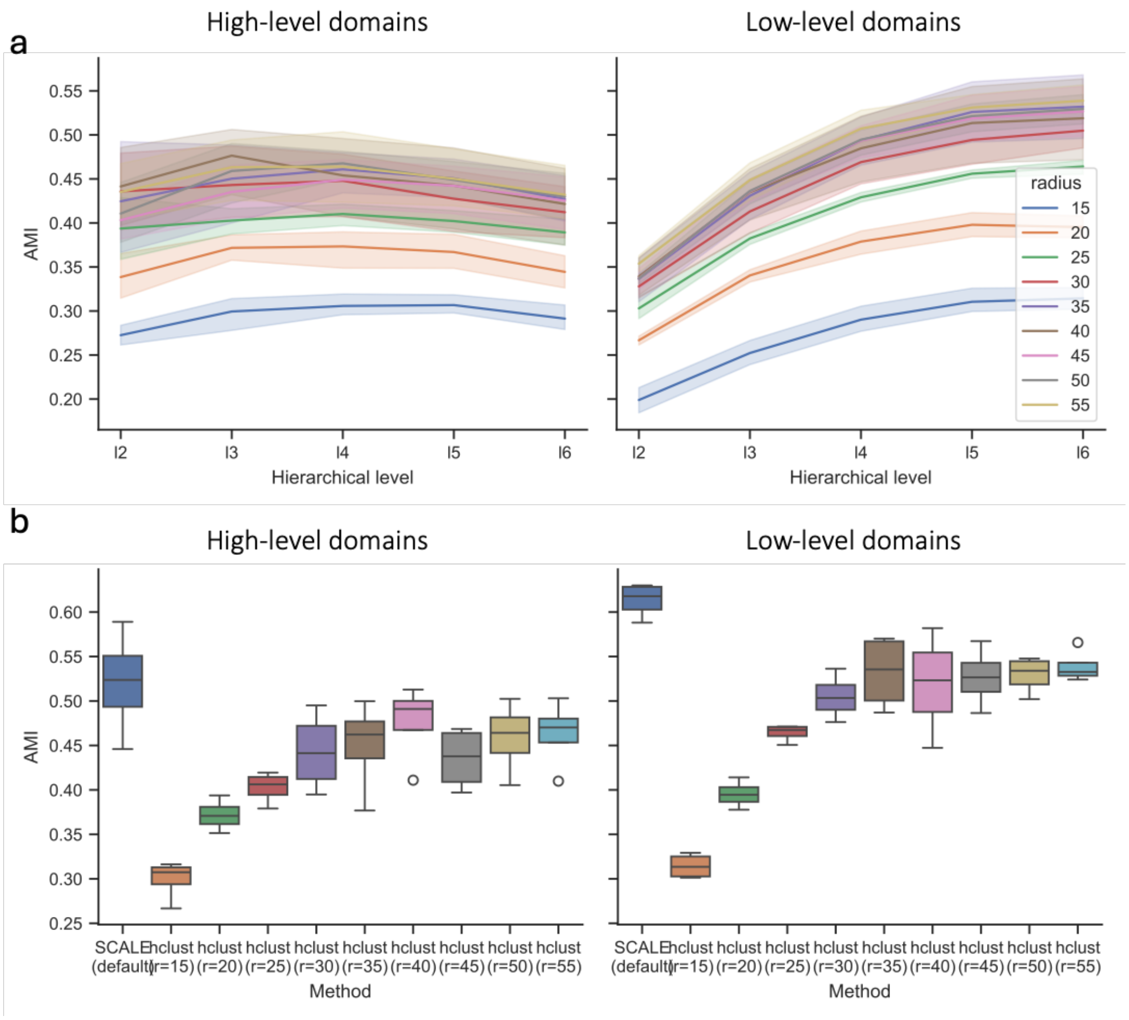


**Supplementary Figure 6.** Performance comparison of SCALE with hierarchical clustering on the Merfish mouse brain dataset in terms of AMI. **(a)** The performance of domain identification against the threshold to cut the hierarchical tree for different radius values. **(b)** Comparison of SCALE and hierarchical clustering for different radius values. For each radius, we cut the hierarchical tree based on the best AMI we obtain.

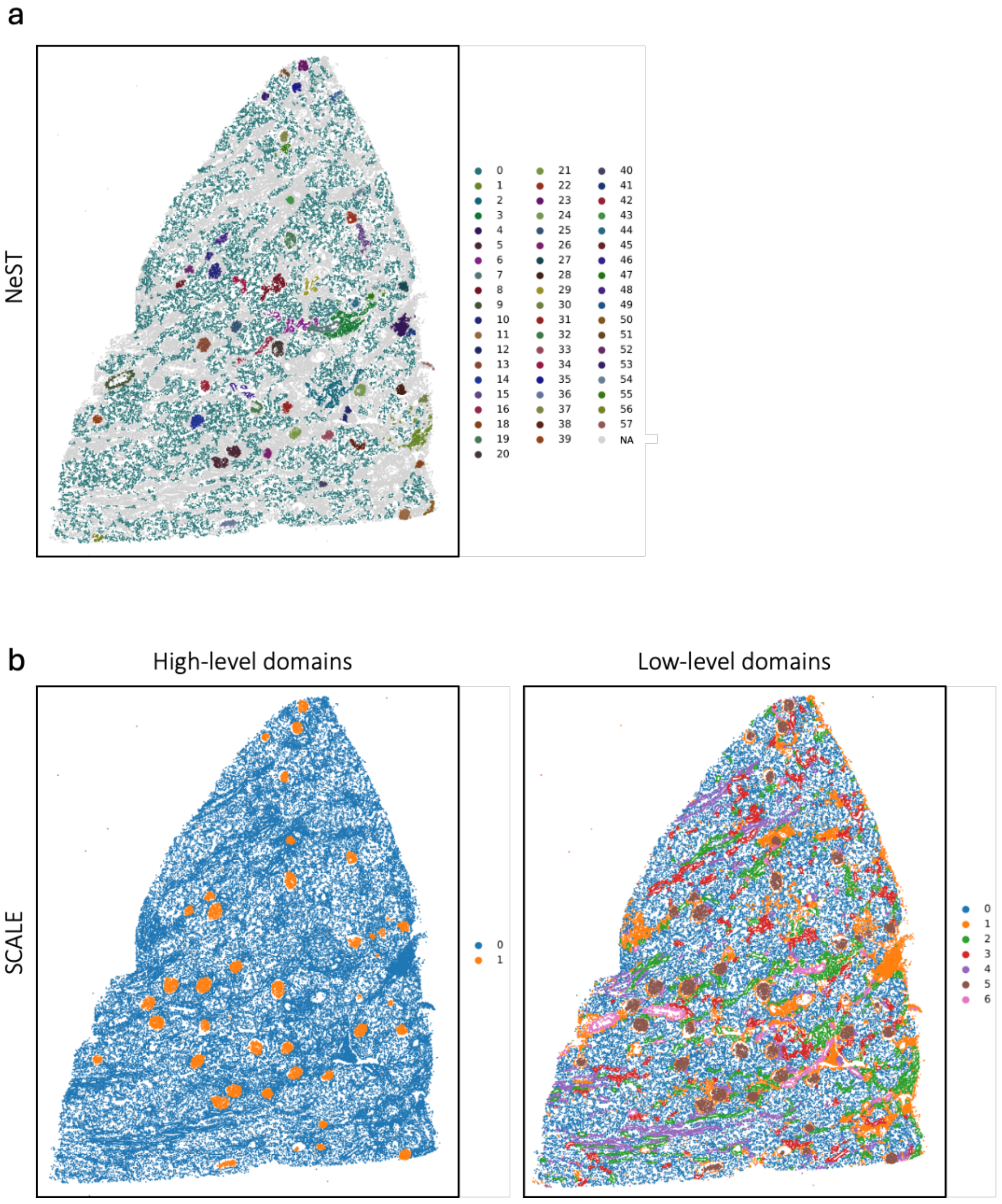


**Supplementary Figure 7.** Clusters identified for the kidney tissue we used in our study **(a)** NeST and **(b)** SCALE at two scales. NeST identifies 58 clusters and an undefined "NA" group, while SCALE identifies two and seven clusters for each scale, respectively.

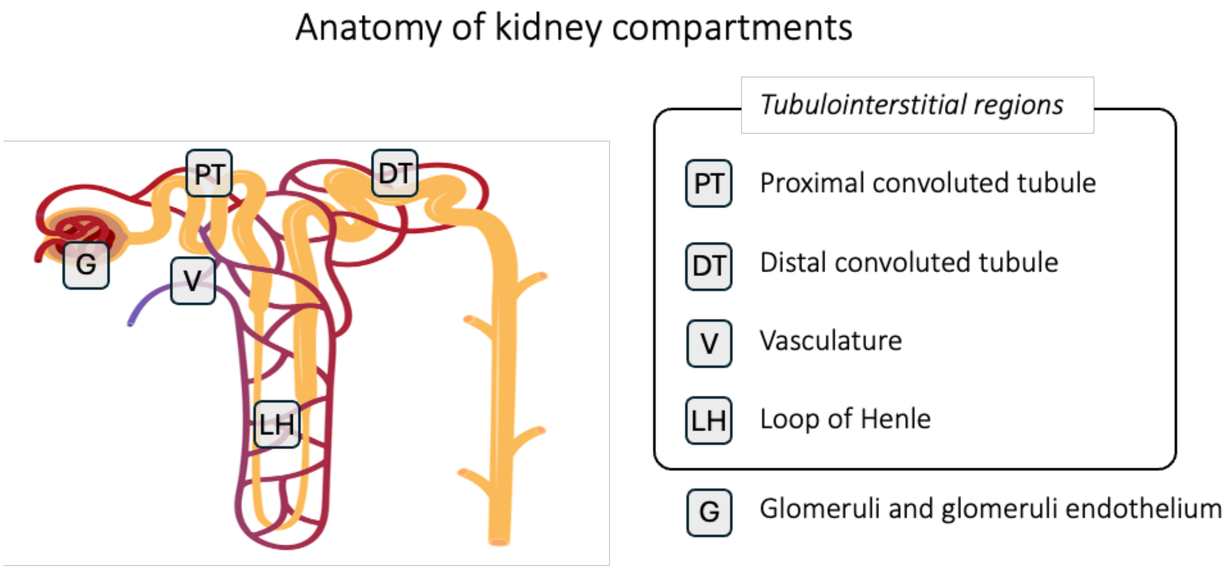


| Domains | Marker genes |
|---|---|
| Glomeruli | NPHS1, NPHS2, SYNPO, CDKN1C, WT1, FOXC2, MAFB, EFNB2, FOXL1, CD2AP, PLCE1, MYH9 |
| Glomeruli endothelium | PLAT, EMCN, TSPAN7, MAPT, KDR, SMAD6, EHD3, FLT1, KDR, BMX |
| Vasculature | NRP1, CDH5, ELN, SMAD6, LPL, FBLN2, EDN1, FBLN5, KLF4, CAS6 |
| Mesangium | SERPINE2, DES, TAGLN |
| Proximal convoluted tubule | SLC34A1, LRP2, HXYD2, HRSP12, ACSM1, ACSM2, ATP11A, CPT1A, NOTCH2, VCAM1, SLC13A3, SLC1A1, SLC5A2, SLC5A12, SLC6A19, ADRA1A |
| Distal convoluted tubule | PVALB, SLC12A3, CALB1, SLC8A1, KLK1WNK1, FXYD2, TRPM7 |

**Supplementary Figure 8.** The anatomy of kidney compartments with the list of marker genes for each compartment. We used this gene list to annotate spatial clusters.

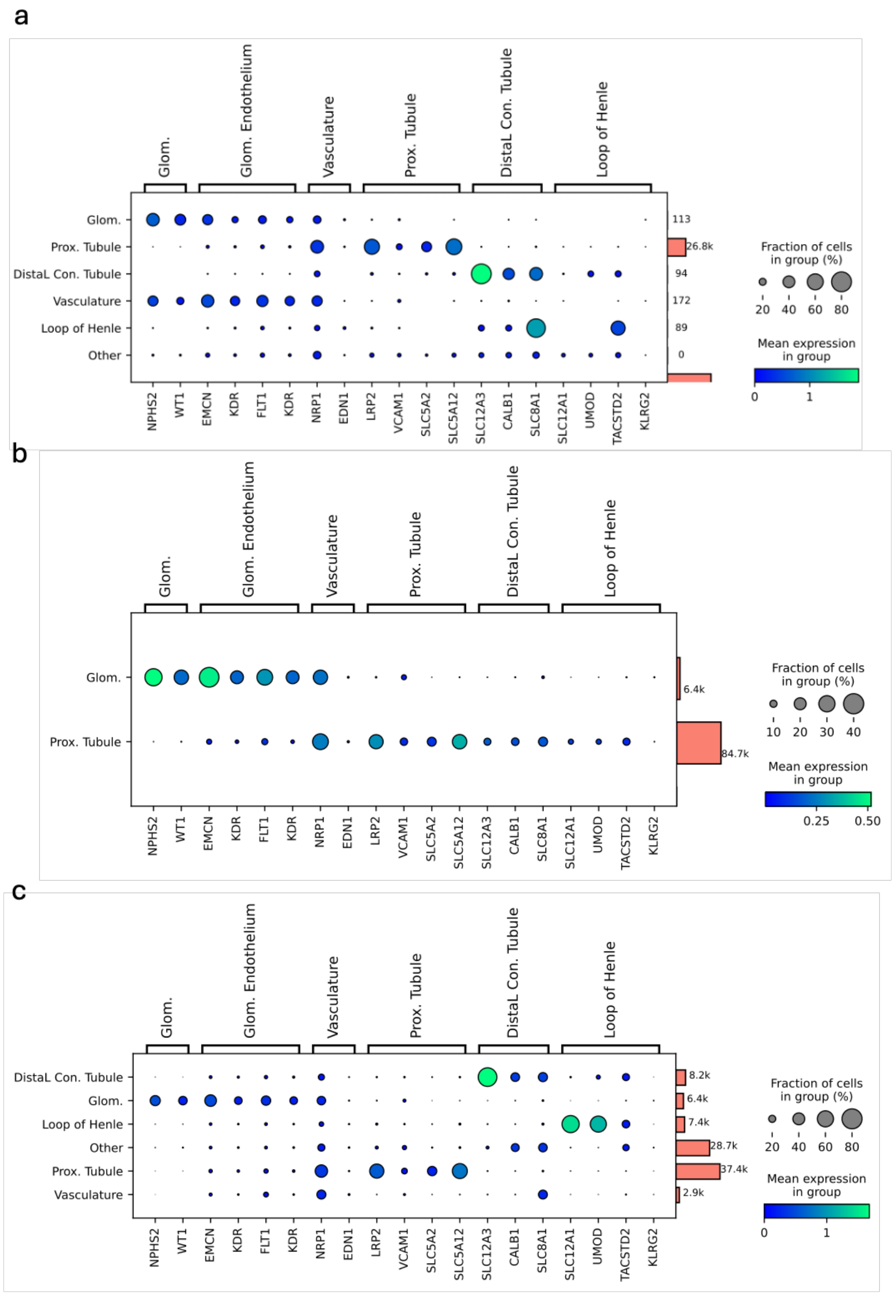


**Supplementary Figure 9.** Dot plots showing the extent of marker gene expression for each annotated compartment. Dot colors indicate mean-normalized expressions of each marker gene within a compartment and dot sizes indicate the percentage of cells within each compartment expressing the respective marker gene. Clusters were identified using **(a)** NeST, **(b)** SCALE at higher resolution, and **(c)** SCALE at lower resolution. The marginal bar plots show the average mean expression per domain.

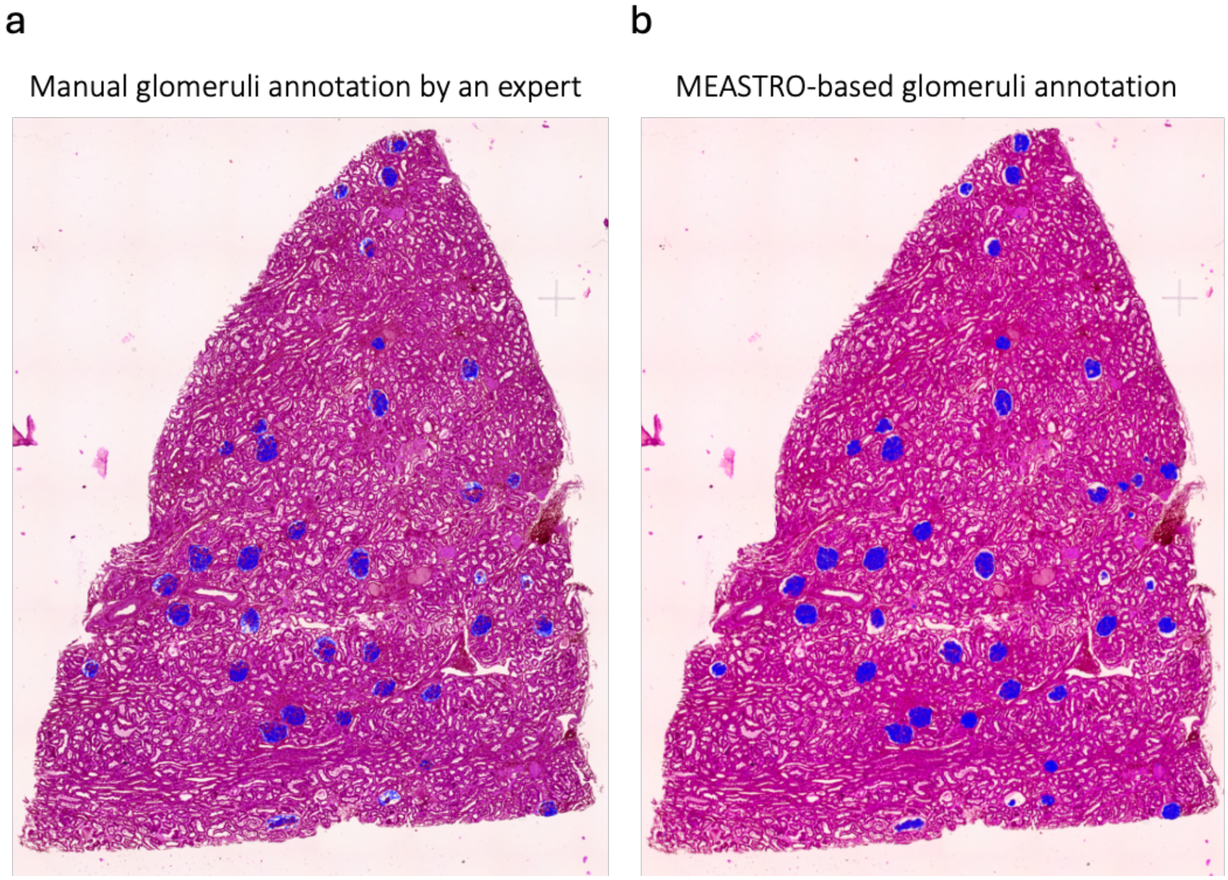


**Supplementary Figure 10.** Hematoxylin and eosin (H&E)-stained image of kidney tissue showing glomeruli annotations by **(a)** a human expert and **(b)** SCALE. Both sets of annotations are shown in blue. The annotated regions exhibit strong alignment, with SCALE achieving a sensitivity of 100% and a specificity of 88% at the glomerulus level.

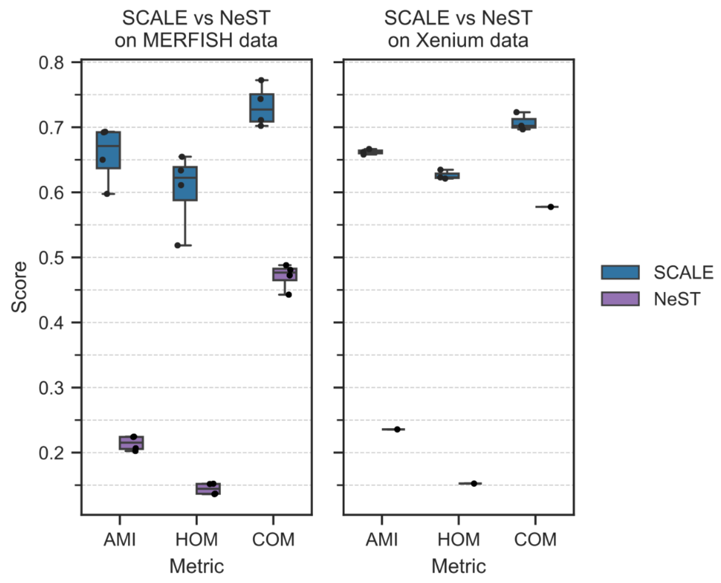


**Supplementary Figure 11.** Performance comparison between SCALE and NeST on the MERFISH (left) and Xenium (right) mouse brain datasets regarding AMI, HOM, and COM scores. Dots show individual sample performance, while the boxplot displays the median, the 25th and 75th percentiles, and whiskers extending 1.5 times the interquartile range. For the Xenium dataset, NeST only identified domains for one sample (for different tested parameter sets).

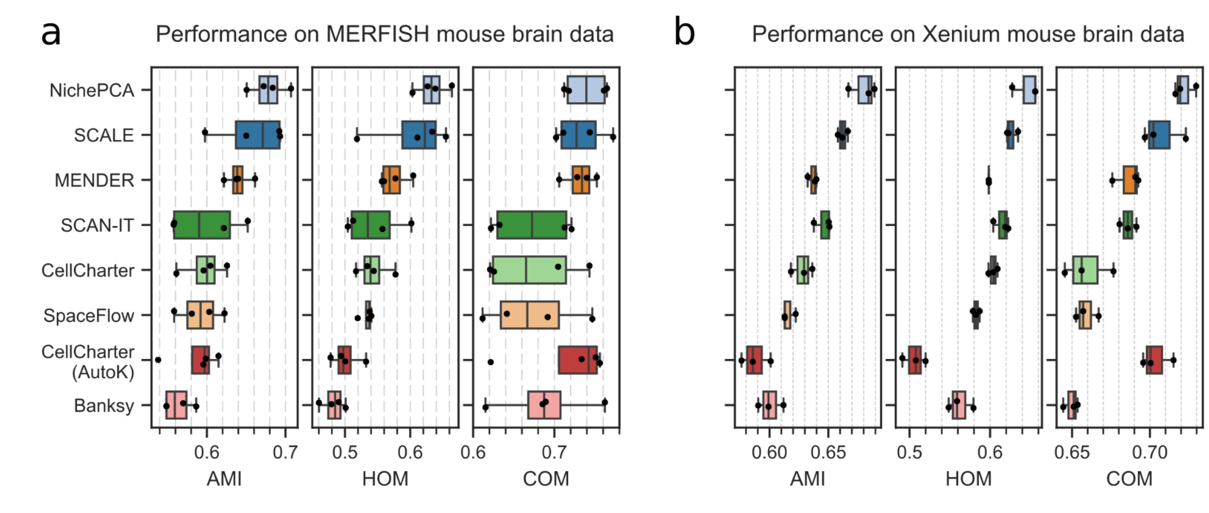


**Supplementary Figure 12.** Spatial domain identification performance for SCALE compared against different methods with supervised hyperparameter selection on Dataset 1 **(a)** and Dataset 5 **(b)** in terms of AMI, HOM, and COM scores. Dots show individual sample performance, while the boxplot displays the median, the 25th and 75th percentiles, and whiskers extending 1.5 times the interquartile range. All results are ordered according to the average AMI score on Dataset 1.

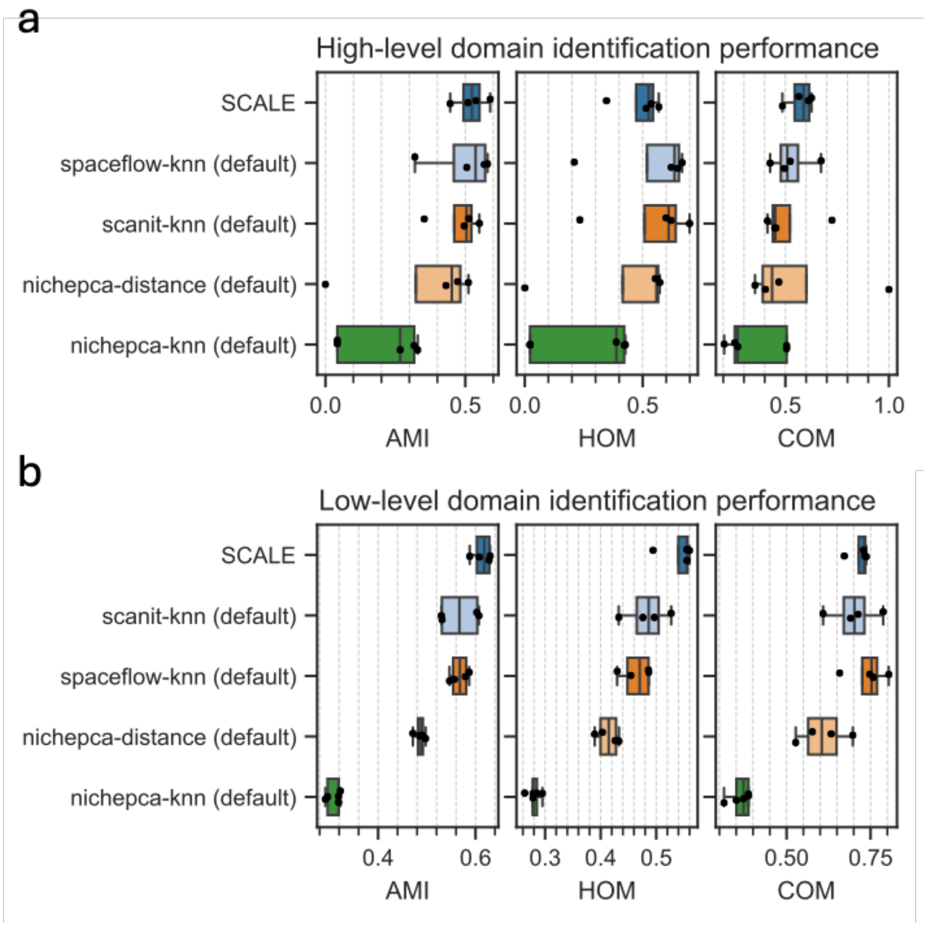


**Supplementary Figure 13.** Multi-sale domain identification performance of SCAN-IT, SpaceFlow, and NichePCA based on a naive approach of randomly sampling two Leiden resolutions per sample on Dataset 1. (a) performance for high-level domains; (b) performance for low-level domains.

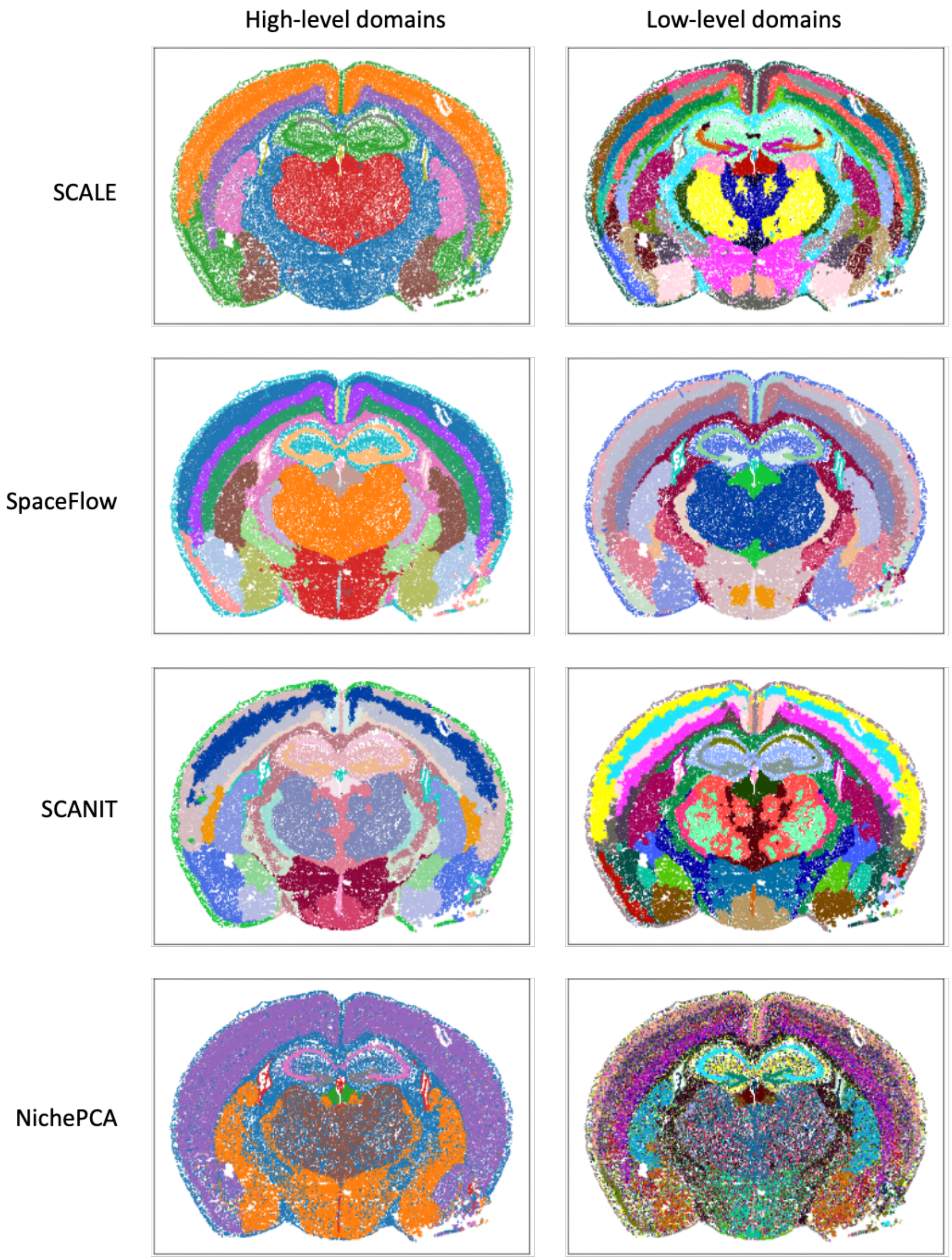


**Supplementary Figure 14.** Domains identified by SCALE in comparison to randomly picked resolutions of SCANIT, SpaceFlow, and NichePCA on Dataset 1. As can be seen, SCALE identifies more coherent spatial domains, while especially SCANIT and NichePCA result in discontinuous, ‘shattered’ clusters.

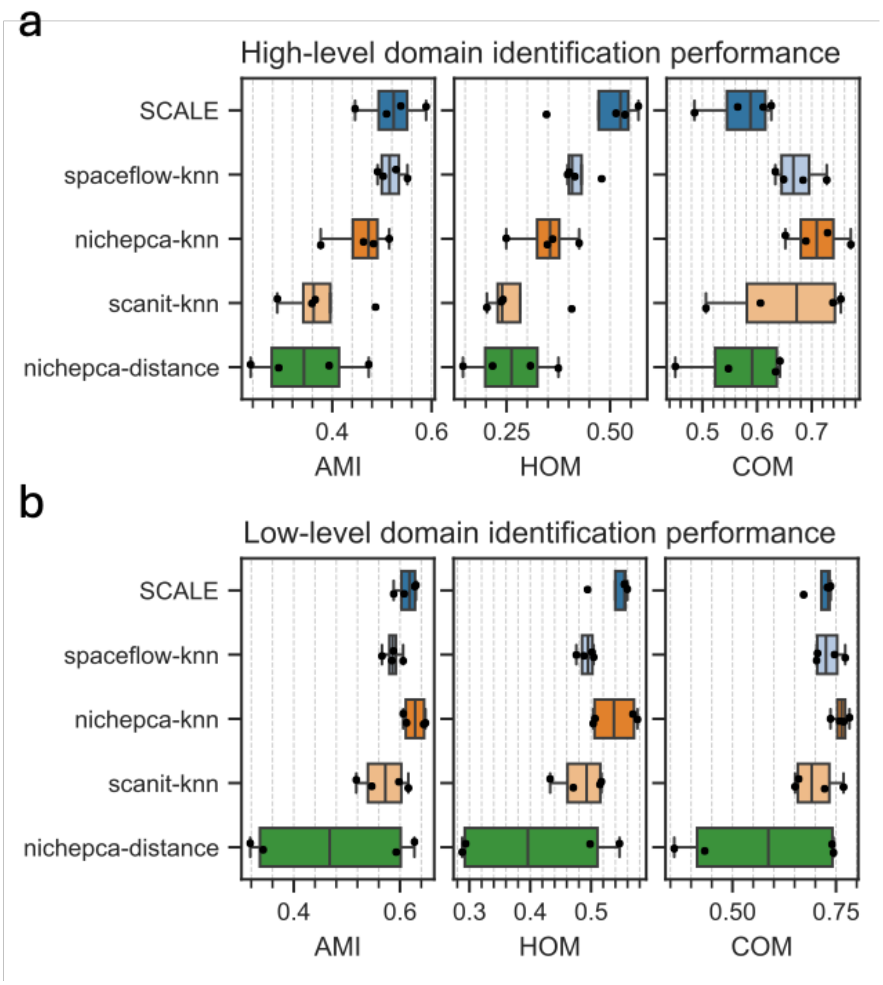


**Supplementary Figure 15.** Multi-sale domain identification performance of SCALE, SCAN-IT, SpaceFlow, and NichePCA based on our proposed scale search algorithm on Dataset 1. (a) performance for high-level domains; (b) performance for low-level domains.

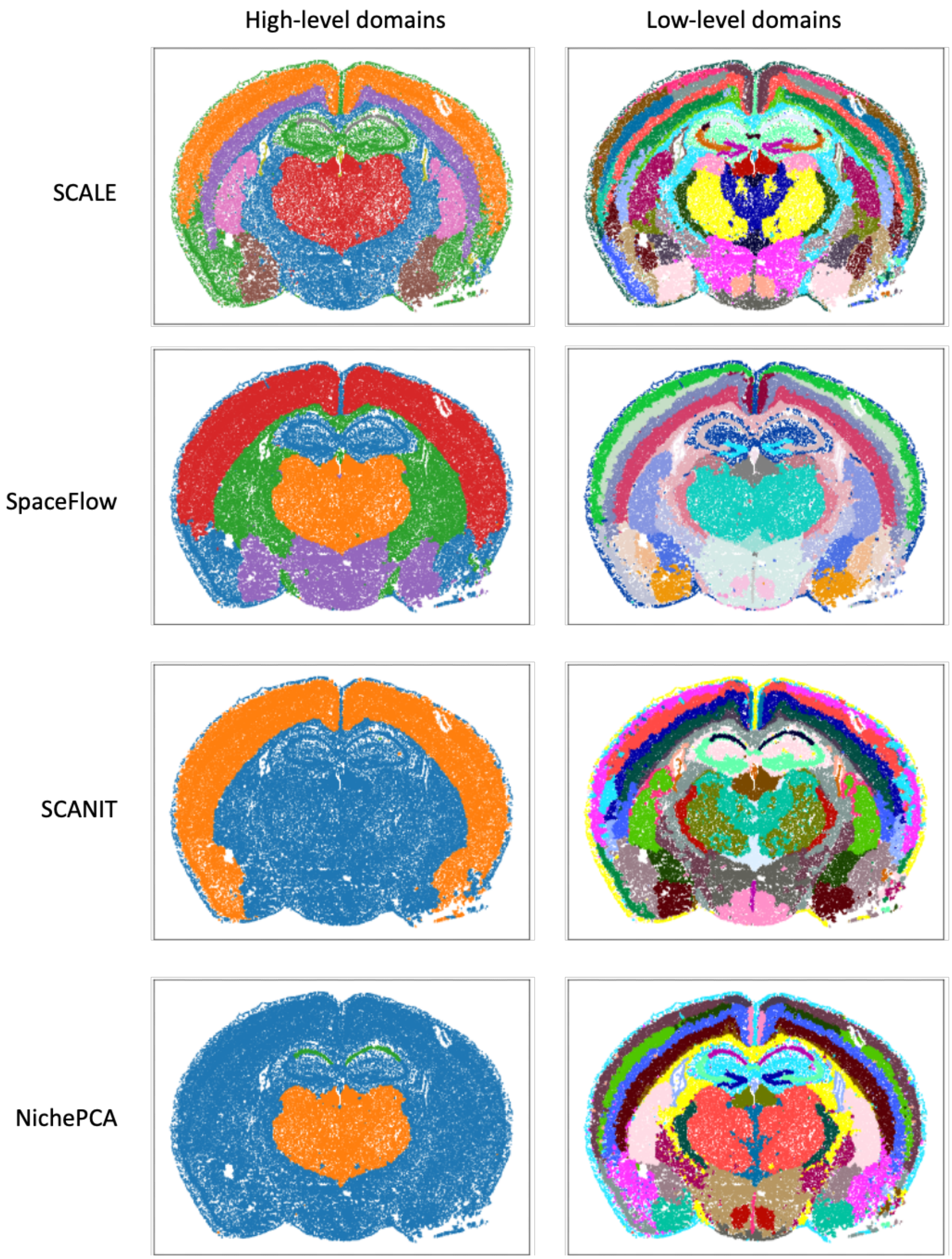


**Supplementary Figure 16.** Domains identified by SCALE, SCANIT, SpaceFlow, and NichePCA based on our proposed scale search algorithm on Dataset 1. While SCALE, Spaceflow and NichePCA show good clustering, SCANIT results in more discontinuous, ‘shattered’ clusters.

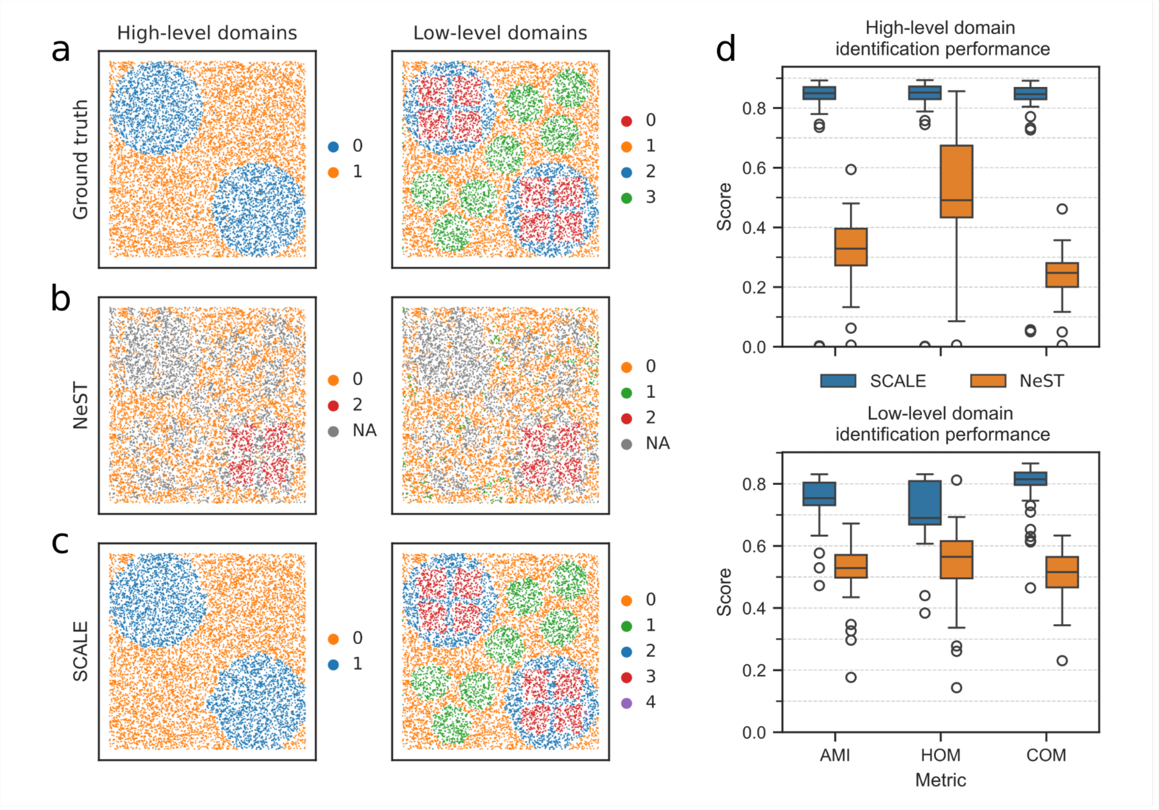


**Supplementary Figure 17.** Multi-scale domain identification results on simulated data. (**a**) Ground truth high-level and low-level domain annotations, (**b**) identified domains by NeST, and (**c**) SCALE for a representative sample from our 50 simulated samples. Cells not assigned to any domain by NeST are labeled as "NA". (**d**) Performance comparison between SCALE and NeST across all 50 simulated samples at high- and low-level scales in terms of AMI, HOM, and COM scores. On one sample, NeST and SCALE assigned all cells to a single domain, leading to near-zero scores.

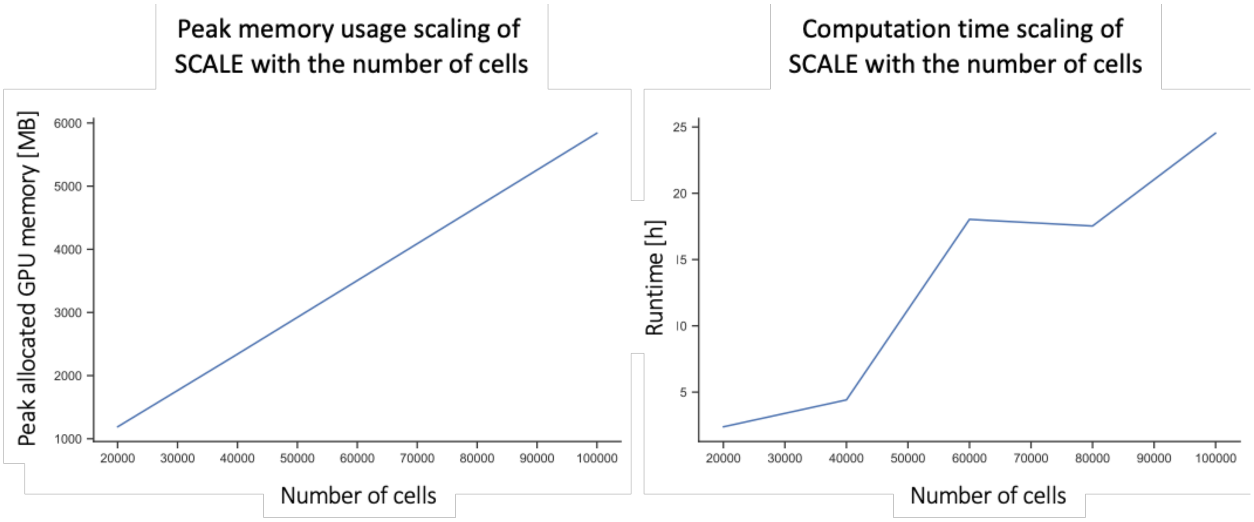


**Supplementary Figure 18.** SCALE runtime and peak memory scaling for an increasing number of cells (20k, 40k, 60k, 80k, and 100k).
